# Repurposing Diabetes Therapies in CKD: Mechanistic Insights, Clinical Outcomes and Safety of SGLT2i and GLP-1 RAs

**DOI:** 10.3390/ph18081130

**Published:** 2025-07-28

**Authors:** Syed Arman Rabbani, Mohamed El-Tanani, Rakesh Kumar, Manita Saini, Yahia El-Tanani, Shrestha Sharma, Alaa A. A. Aljabali, Eman Hajeer, Manfredi Rizzo

**Affiliations:** 1 RAK College of Pharmacy, RAK Medical and Health Sciences University, Ras Al Khaimah 11172, United Arab Emirates; arman@rakmhsu.ac.ae; 2 Amity Institute of Pharmacy, Amity University, Gurgaon 122413, Haryana, India; 3 Department of Pharmacy, Jagannath University, Bahadurgarh 124507, Haryana, India; 4 Geeta Institute of Pharmacy, Geeta University, Panipat 132145, Haryana, India; 5 Royal Cornwall Hospital, NHS Trust, Truro TR1 3LJ, UK; 6 Department of Pharmaceutics and Pharmaceutical Technology, Faculty of Pharmacy, Yarmouk University, Irbid 21163, Jordan; 7 Faculty of Biology, Medicine and Health, University of Manchester, Manchester M13 9PL, UK; 8 Department of Health Promotion, Mother and Childcare, Internal Medicine and Medical Specialties, School of Medicine, University of Palermo, 90127 Palermo, Italy

**Keywords:** (CKD) Chronic Kidney Disease, (DKD) Diabetic Kidney Disease, SGLT2i, GLP-1 RAs

## Abstract

**Background:** Chronic Kidney Disease (CKD) is a major global health issue, with diabetes being its primary cause and cardiovascular disease contributing significantly to patient mortality. Recently, two classes of medications—sodium–glucose cotransporter 2 inhibitors (SGLT2i) and glucagon-like peptide-1 receptor agonists (GLP-1 RAs)—have shown promise in protecting both kidney and heart health beyond their effects on blood sugar control. **Methods:** We conducted a narrative review summarizing the findings of different clinical trials and mechanistic studies evaluating the effect of SGLT2i and GLP-1 RAs on kidney function, cardiovascular outcomes, and overall disease progression in patients with CKD and DKD. **Results:** SGLT2i significantly mitigate kidney injury by restoring tubuloglomerular feedback, reducing intraglomerular hypertension, and attenuating inflammation, fibrosis, and oxidative stress. GLP-1 RAs complement these effects by enhancing endothelial function, promoting weight and blood pressure control, and exerting direct anti-inflammatory and anti-fibrotic actions on renal tissues. Landmark trials—CREDENCE, DAPA-CKD, and EMPA-KIDNEY—demonstrate that SGLT2i reduce the risk of kidney failure and renal or cardiovascular death by 25–40% in both diabetic and non-diabetic CKD populations. Likewise, trials such as LEADER, SUSTAIN, and AWARD-7 confirm that GLP-1 RAs slow renal function decline and improve cardiovascular outcomes. Early evidence suggests that using both drugs together may offer even greater benefits through multiple mechanisms. **Conclusions:** SGLT2i and GLP-1 RAs have redefined the therapeutic landscape of CKD by offering organ-protective benefits that extend beyond glycemic control. Whether used individually or in combination, these agents represent a paradigm shift toward integrated cardiorenal-metabolic care. A deeper understanding of their mechanisms and clinical utility in both diabetic and non-diabetic populations can inform evidence-based strategies to slow disease progression, reduce cardiovascular risk, and improve long-term patient outcomes in CKD.

## 1. Introduction

### 1.1. Epidemiology of CKD and DKD

CKD affects over 700 million people worldwide—approximately 9% of the global population—with nearly 4 million requiring kidney replacement therapy (KRT) [1]. While women are more likely to develop early-stage CKD, men have a higher risk of progressing to end-stage kidney disease (ESKD). DKD is the leading cause of CKD and ESKD, accounting for 50% of cases globally [2].

DKD significantly reduces quality of life and increases cardiovascular mortality. The UK Prospective Diabetes Study found that after 15 years, 28% of patients with type 2 diabetes had impaired renal function, and 38% had albuminuria [3]. Common contributors to CKD in type 2 diabetes include hypertension, dyslipidemia, obesity, AKI, glomerular atherosclerosis, and age-related decline [4].

In the U.S., one in three adults have diabetes and one in seven have CKD [5]. In Europe, CKD is 2–5 times more prevalent in individuals with type 2 diabetes compared to those without [6]. Notably, 90% of hospitalized CKD patients have hypertension, and 16% have diabetes mellitus. The case fatality rate is 21% in CKD and rises to 51% in ESKD [7].

### 1.2. Conventional Therapeutic Methods and Their Challenges

Early diagnosis of CKD is critical, especially in patients with diabetes, hypertension, or a family history of kidney disease. Routine testing using urine albumin-to-creatinine ratio (UACR) and estimated glomerular filtration rate (eGFR) enables early detection [8]. Renin–angiotensin–aldosterone system (RAAS) inhibitors, including ACE inhibitors (enalapril, lisinopril, ramipril) and ARBs (losartan, valsartan, Irbesartan), remain the cornerstone for slowing CKD progression—particularly in proteinuric patients [7]. Mineralocorticoid receptor antagonists (MRAs) like spironolactone and eplerenone, and the newer nonsteroidal MRA finerenone, offer added anti-inflammatory and anti-fibrotic benefits [9]. However, disease progression often continues despite treatment.

RAAS inhibitors carry risks of hyperkalemia and acute kidney injury (AKI), particularly in susceptible patients or when used with other antihypertensives [10,11]. Their use in advanced CKD is limited due to reduced efficacy and safety concerns. Discontinuation may increase cardiovascular risk, highlighting the delicate balance in long-term therapy [12].

For end-stage kidney disease (ESKD), dialysis supports fluid and waste removal but cannot fully replicate renal endocrine functions like erythropoietin synthesis and vitamin D activation [9]. Complications include malnutrition, infection, and cardiovascular strain. In many low- and middle-income countries, access remains limited due to high cost and infrastructure constraints [12]. By 2030, the global population requiring kidney replacement therapy (KRT) is projected to more than double to 5.4 million, with Asia accounting for the largest growth—from 1 million to an estimated 2 million patients [13].

Using SGLT2i (SGLT2i), such as dapagliflozin, empagliflozin, and canagliflozin, targets novel renal and cardiovascular pathways not fully addressed by conventional therapies like dialysis or RAAS blockers [14]. These agents act by inhibiting sodium–glucose cotransporter-2 in the proximal tubules, reducing glucose and sodium reabsorption, thereby restoring tubuloglomerular feedback and lowering intraglomerular pressure—a key driver of DKD progression [15]. They also reduce blood pressure, promote modest weight loss, alleviate inflammation and oxidative stress, and improve glycemic control independently of insulin. Major trials—CREDENCE, CANVAS, and EMPA-REG—have consistently demonstrated their cardiorenal protective benefits, including in non-diabetic CKD patients [16].

GLP-1 receptor agonists (GLP-1 RAs), including liraglutide, semaglutide, and dulaglutide, mimic endogenous GLP-1, enhancing glucose-dependent insulin secretion, delaying gastric emptying, suppressing glucagon, and increasing satiety—contributing to glycemic control and weight loss [17]. Trials like LEADER and SUSTAIN-6 have shown that GLP-1 RAs lower cardiovascular risk and slow DKD progression [18]. Additionally, they improve endothelial function, reduce albuminuria, and exert anti-inflammatory and anti-fibrotic effects on the kidneys [14].

Together, SGLT2i and GLP-1 RAs offer complementary mechanisms that modulate hemodynamic, metabolic, and inflammatory pathways, providing enhanced protection against renal and cardiovascular deterioration. When combined with standard RAAS inhibition, this multimodal approach strengthens defense against DKD progression and its complications [12].

Ongoing studies aim to further define their roles in non-diabetic CKD, potentially expanding indications across broader patient populations [8]. Given CKD’s growing global burden, therapies that go beyond glucose lowering to offer renal and cardiovascular benefits are essential [18]. This review will discuss the mechanisms, clinical benefits and potential integration of these novel therapies into CKD care [14]. By integrating these therapies into standard CKD care, healthcare providers may achieve better renal outcomes, reduced dialysis dependency, improved overall patient health, and optimize their use and benefits in non-diabetic CKD populations [19].

## 2. Methodology

This review was conducted using a narrative literature review methodology. Relevant studies were identified through a systematic search of electronic databases, including PubMed, Scopus, Embase and Cochrane, focusing on publications on SGLT2i and GLP-1RA in mechanistic insights of CKD and DKD from inception to March 2025. Keywords used for searching databases: SGLT2i, GLP-1 RAs, DKD, CKD, renal outcomes, cardiovascular outcomes and clinical trials. Emphasis was placed on including landmark randomized controlled trials, meta-analyses, and real-world studies relevant to the management of CKD and DKD. Additionally, to ensure comprehensive coverage, reference lists of all eligible studies were manually screened to identify further relevant articles not captured in the initial database search.

## 3. Pathophysiology of Kidney Disease: Targets for SGLT2i and GLP-1 RA

### 3.1. Key Pathways in Kidney Disease Progression

**Figure 1:**
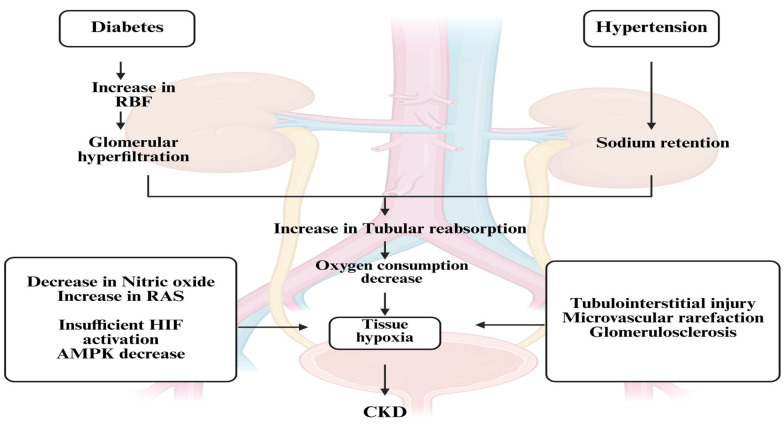
Pathophysiology of CKD and DKD. Description: This diagram illustrates the multifactorial mechanisms underlying CKD and DKD, including hemodynamic changes, metabolic disturbances, inflammation, oxidative stress and fibrosis. These interconnected pathways contribute to progressive renal injury, albuminuria, and declining glomerular filtration rate, ultimately leading to end-stage kidney disease. The figure highlights key molecular targets and cellular processes involved in disease progression.

CKD represents a gradual loss of renal function over time, driven by a complex interplay of metabolic, hemodynamic, inflammatory, and fibrotic mechanisms [15]. Multiple molecular and cellular pathways contribute to the progression of kidney injury, irrespective of the initial cause. A deeper understanding of these pathways is crucial for identifying therapeutic targets and improving outcomes in patients with CKD. Renal damage is accelerated by the interaction of four major pathways: oxidative stress, fibrosis, inflammation and hyperfiltration [20]. The primary causes of DKD in individuals with type 1 diabetes are glomerular hyperfiltration and chronic hyperglycemia. However, because a number of cardiovascular risk factors including obesity, dyslipidemia and hypertension may also play a role in the development of microvascular damage [21]. The pathogenesis of DKD in individuals with type 2 diabetes is more complicated, as shown in Figure 1.

#### 3.1.1. Hemodynamic Factors in DKD and CKD: The Role of Glomerular Hyperfiltration

The kidney features a unique double capillary system, with glomerular pressure regulated by the balance of tone between the afferent and efferent arterioles [14]. In diabetes, this balance is often disrupted, leading to glomerular hyperfiltration—an early and critical factor in the development and progression of DKD. Hyperfiltration is typically defined as a glomerular filtration rate (GFR) between 120 and 180 mL/min/1.73 m^2^, or a GFR exceeding two standard deviations above the mean for age-matched healthy individuals [7]. It affects approximately 70% of patients with type 1 diabetes (T1DM) and 50% with type 2 diabetes (T2DM) within the first 1–5 years of diagnosis [19,22].

This elevated intraglomerular pressure predisposes nephrons to structural damage, contributing to long-term nephron loss. Mechanistically, glucose reabsorption in the proximal tubule reduces sodium chloride delivery to the macula densa, impairing tubuloglomerular feedback [23]. As a compensatory response, angiotensin II-mediated vasoconstriction of the efferent arteriole further increases glomerular pressure, exacerbating hyperfiltration. Over time, this leads to mesangial and podocyte stretching, glomerulomegaly, and progressive glomerular injury. In T2DM, the pathology is more heterogeneous and can be categorized into three main classes [24,25]:

- Class I represents typical diabetic glomerulopathy.
- Class II presents with relatively preserved glomerular structure but prominent vascular and interstitial changes, often showing early GFR decline without albuminuria.
- Class III includes subclasses IIIa (19% of cases) and IIIb (18%), both lacking significant glomerular basement membrane (GBM) thickening and mesangial expansion. Patients in this group may also experience silent episodes of acute kidney injury (AKI), contributing to gradual functional decline [20].

#### 3.1.2. Changes in Endothelial Cells

Exposure of endothelial cells to high glucose levels activates the polyol pathway, leading to increased reactive oxygen species (ROS) production and mitochondrial dysfunction [26]. This oxidative stress upregulates adhesion molecules, promoting immune cell recruitment and inflammation. In the kidneys, glomerular endothelial cells (GEnCs) line the glomerular capillaries and play a vital role in maintaining filtration barrier integrity [27]. These cells are coated with a glycocalyx, a protective layer of polysaccharides that helps regulate vascular permeability. Loss of this glycocalyx, as seen in diabetic models, is closely linked to the development of albuminuria. Animal studies have shown that mitochondrial damage in GEnCs contributes to increased permeability, podocyte injury, proteinuria, and glomerulosclerosis [28]. However, mitochondria-targeted antioxidants can effectively prevent oxidative stress in GEnCs, preserving fenestrations and protecting against glycocalyx degradation, ultimately reducing endothelial dysfunction and albumin leakage [26].

#### 3.1.3. Dyslipidemia

A significantly elevated risk of cardiovascular disease is common in patients with CKD. Patients with CKD consistently experience dyslipidemia, which is defined by elevated serum triglycerides, low HDL cholesterol (HDL-C) and LDL-cholesterol (LDL-C) levels [29]. The primary cause of death for people with eGFR < 60 mL/min per 1.73 m^2^ is cardiovascular disease (CVD). Dyslipidemia in nephrotic syndrome (NS) is characterized by elevated LDL and VLDL levels and normal HDL levels [24]. Recent post hoc and meta-analyses of clinical trial data provide evidence that abnormal lipids are linked to a greater loss of GFR and that statin medication may help with both the progression of CKD and the risk of cardiovascular disease [30]. Statins may even delay the course of stage 3 CKD patients. It seems that statins’ positive effects go beyond just decreasing cholesterol [31].

#### 3.1.4. Abnormal Angiogenesis

By preferentially widening the efferent arteriole, ACEIs lower glomerular capillary pressure probably due to the suppression of angiotensin II (AngII) [32]. In fact, most experimental research have shown that angiotensin type 1 receptor blockers (ARBs), which lack the ability to enhance bradykinin, do not significantly dilate the efferent arteriole or lower glomerular pressures to the same degree as ACEIs [33]. The combined usage of ARBs and ACEIs appears to have a higher effect in reducing proteinuria which is not related to effects on systemic blood pressure. The protein LRG1, which is primarily produced by GEnCs, enhances endothelial transforming growth factor/activin receptor-like kinase 1 signaling which contributes to angiogenesis and the pathophysiology of DKD [34]. Global LRG1 mutations reduced glomerular angiogenesis, oxidative damage and provided protection against DKD [31].

#### 3.1.5. Podocytes Dysfunction in DKD

Podocytes form the epithelial lining of the glomerulus and are essential for the selective filtration of molecules smaller than 60 kDa. Damage or loss of podocyte foot processes is closely associated with proteinuria and the development of nephrotic syndrome, particularly in advanced stages of DKD [35]. In early DKD, podocyte foot process effacement and widening occur, while significant podocyte loss (>20%) marks an irreversible phase, leading to glomerular scarring and end-stage renal disease (ESRD) [21]. While partial inhibition of the mTOR pathway in podocytes has shown therapeutic benefits, complete loss of mTORC1 activity can worsen kidney damage and promote glomerulosclerosis. In addition to mTOR, podocyte hypertrophy is regulated by key energy-sensing pathways involving LKB1 and AMP-activated protein kinase (AMPK), highlighting their role in maintaining podocyte structure and function under diabetic stress [25].

#### 3.1.6. Specific Cytokines/Growth Factors and Progression of CKD

A range of cytokines and growth factors contribute to the development of glomerular and tubulointerstitial scarring in kidney disease, with their roles varying across different stages of injury [36]. Key mediators include TGF-β, PDGF, Angiotensin II (AngII), basic FGF, endothelin, chemokines, PAI-1, and PPAR-γ, which influence fibrosis through altered gene expression and signaling pathways. Among these, TGF-β is the central driver of renal fibrosis, promoting extracellular matrix (ECM) accumulation. It also induces the expression of PAI-1 and AngII, both of which contribute to fibrotic progression [37]. Elevated PAI-1 levels are associated with both renal and cardiovascular fibrosis. In animal models, transgenic overexpression of TGF-β leads to progressive kidney damage, whereas blocking TGF-β or PDGF-B reduces mesangial matrix expansion, as seen in the anti-Thy1 model [38]. Interestingly, TGF-β deficiency can lead to immune dysregulation and lymphoproliferative disorders, highlighting its dual role in fibrosis and immune modulation. At low concentrations, TGF-β supports podocyte growth arrest and differentiation, indicating its context-dependent effects in kidney pathology [39].

### 3.2. Mechanistic Rationale for SGLT2 Inhibition

SGLT2i was approved to treat type 2 diabetes and developed into a powerful cardio- and renoprotective tool in the management of heart failure and type 2 diabetes [40]. More recently, they have emerged as an intriguing stand-alone treatment option for CKD, independent of the presence of type 2 diabetes. It all began in 1835 when a French chemist developed phlorizin, a natural substance used to cure fever and infectious disorders [41]. By blocking SGLT2, the tubuloglomerular feedback is returned to normal, and the reabsorption of glucose and sodium is reduced. The connection between SGLT2 and Na^+^/H^+^ exchanger 3 (NHE3) is another important component. The natriuretic impact of SGLT2i is further explained by the reduction in NHE3 that has been found in multiple investigations [39,41].

#### 3.2.1. Proximal Tubule Cell Pathology Correlates with SGLT2

A modest decline in the projected glomerular filtration rate can also be explained by the proximal tubule cell pathway [42]. Remarkably, dapagliflozin treatment reduces the filtration fraction without raising renal vascular resistance. It suggests that post-glomerular vasodilation (not pre-glomerular vasoconstriction) is the cause of SGLT2 inhibition’s lowering outcome for glomerular filtration [43] and explains the renoprotective benefits regardless of the existence of diabetes mellitus by making the well-known SGLT2i action (TGF and glucose-independent) [44].

#### 3.2.2. Anti-Inflammatory and Anti-Fibrotic Responses via SGLT2

Beyond glucose control, SGLT2i exhibit notable anti-inflammatory, anti-fibrotic, and anti-hypoxic effects, contributing significantly to their renal and cardiovascular protective benefits in CKD and diabetic nephropathy [45]. These pleiotropic actions are increasingly recognized as key mechanisms behind the favorable outcomes seen in clinical trials. In preclinical studies, phlorizin-mediated SGLT inhibition increased oxygen levels in the renal cortex but reduced medullary pO_2_, possibly due to a shift in active transport to the distal nephron [46]. Separately, animal models have shown that dapagliflozin reduces renal injury by enhancing the expression of hypoxia-inducible factor 1 (HIF-1) and related protective proteins in hypoxic kidney cells, suggesting a direct role in mitigating tubulointerstitial hypoxia [47].

#### 3.2.3. Metabolic Benefits (Glycemic Control)

SGLT2-mediated glucose uptake in proximal tubular cells is a key driver of oxidative stress and inflammation, contributing to tubular hypoxia and kidney injury [46]. By blocking this pathway, SGLT2i not only improve oxygenation but also reduce cellular stress and injury. Studies in diabetic models have shown that SGLT2 inhibition lowers oxidative, inflammatory, and fibrotic responses—partly by suppressing the AGE–RAGE pathway, a pro-apoptotic cascade triggered by oxidative damage [37]. Moreover, SGLT2i have demonstrated dose-dependent reductions in mesangial expansion, macrophage infiltration, and interstitial fibrosis [44]. These renoprotective effects arise through three primary mechanisms i.e., reduction in hyperfiltration and glomerular stress (potentially independent of glucose levels); improvement in hypoxia (which is strongly glucose-dependent); suppression of inflammatory, fibrotic and apoptotic pathways [48]. Together, these actions explain the broad kidney-protective benefits of SGLT2i, making them effective in managing CKD in both diabetic and non-diabetic populations [14].

### 3.3. Mechanistic Rationale for GLP-1 Receptor Activation

Proglucagon is converted into the 30-amino acid peptide hormone known as glucagon-like peptide-1 (GLP-1), that is mostly produced by enteroendocrine L cells of the intestinal epithelium and by tiny clusters of neurons in the brain stem’s tractus solitarius nucleus. GLP-1, which is produced in a nutrient-dependent way, reduces postprandial glucose rise by inducing satiety, inhibiting glucagon release and stomach emptying, and stimulating insulin secretion [38]. The so-called incretin effect is significantly diminished or nonexistent in patients with type 2 diabetes (T2DM) compared to healthy participants, which makes the GLP-1 receptor (GLP-1R) a desirable target for antidiabetic management with GLP-1RAs. Additionally, GLP-1RAs are a novel pharmacological anti-obesity therapeutic approach and result in notable weight loss [48,49].

#### 3.3.1. Anti-Inflammatory, Anti-Fibrotic, and Renoprotective Signaling

GLP-1RAs provide notable cardiovascular and renal benefits in patients with type 2 diabetes, particularly those at high cardiovascular risk [50]. Beyond glucose control, they reduce albuminuria and modestly slow the decline in estimated glomerular filtration rate (eGFR), suggesting unique renoprotective effects. GLP-1 is secreted in two phases post-meal—a rapid surge within 15–30 min and a second peak at 90–120 min—regulated by gut-derived neurotransmitters like acetylcholine and gastrin-releasing peptide [38]. This biphasic release is linked to proximal and distal L-cell activation as nutrients transit through the gut. However, native GLP-1 has a short half-life (<2 min) due to rapid cleavage by DPP-IV enzymes, forming inactive GLP-1^(9−36/9−37)^ with low receptor affinity. Both active and inactive forms are quickly cleared by the kidneys, and only 10–15% of GLP-1 reaches systemic circulation. In patients with renal impairment, GLP-1 clearance is delayed, though its initial enzymatic degradation remains unaffected. These dynamics highlight the complexity of GLP-1 biology and its therapeutic potential in renal disease [51,52].

#### 3.3.2. Indirect Effects via Glycemic/Weight Control and Direct Renal Actions

The renoprotective effects of GLP-1RAs are largely attributed to their ability to reduce inflammation, oxidative stress, promote natriuresis, and lower intraglomerular pressure. In early DKD, elevated systemic oxidative stress plays a critical role in disease progression [53]. Recombinant human GLP-1 has been shown to decrease oxidative damage by inhibiting protein kinase C and activating protein kinase A (PKA) in glomeruli and glomerular endothelial cells [54]. GLP-1RAs also suppress the expression of proinflammatory cytokines, adhesion molecules, and profibrotic mediators. Notably, exenatide reduced reactive oxygen species and inflammatory signaling pathways, such as NF-κB, TNF-α, IL-1β, and TLR4, in obese diabetic patients, independent of weight loss [52,55].

GLP-1-induced natriuresis and diuresis are linked to reduced activity of the Na^+^/H^+^ exchanger 3 (NHE3) in proximal tubules, mediated by PKA-dependent phosphorylation. Lower NHE3 activity enhances sodium delivery to the macula densa, triggering afferent arteriolar vasoconstriction and reducing glomerular hyperfiltration. Clinically, agents like liraglutide initially lower eGFR before stabilization, reflecting their role in modulating renal hemodynamics [48].

## 4. SGLT2i in Kidney Disease

### 4.1. Pharmacological Overview

In healthy individuals, the kidneys play a vital role in glucose homeostasis by reabsorbing nearly all filtered glucose in the proximal tubule, specifically in the S1 to S3 segments. SGLT2, located in the S1/S2 segments, handles the majority of glucose reabsorption, while SGLT1 in the S3 segment reabsorbs the remainder [48,56]. Glucose uptake into tubular cells is driven by the Na^+^ electrochemical gradient, and it exits into circulation via GLUT2 on the basolateral membrane [49]. Sodium is actively transported out of the cell by Na^+^/K^+^-ATPase, maintaining the gradient. SGLT2 transports one Na^+^ per glucose, while SGLT1 uses two Na^+^ ions per glucose molecule [49,57].

Despite high plasma levels, SGLT2i, including empagliflozin, dapagliflozin, and canagliflozin, blocks only 40–50% of glucose reabsorption, as the distal nephron compensates for sodium loss. Glucose-coupled sodium transport contributes to just 10% of total proximal sodium reabsorption, limiting the natriuretic effect of SGLT2 inhibition at therapeutic doses. These agents offer a unique, insulin-independent mechanism of action, making them valuable in managing type 2 diabetes and CKD [37,38].

#### Mechanism of Action

- Inhibition of Renal Glucose Reabsorption

These drugs specifically block the SGLT2 protein, which is mostly expressed in the kidney’s early proximal convoluted tubule. Normally, about 90% of the glucose that the glomeruli filter is reabsorbed by SGLT2 [33]. When SGLT2 is inhibited, this reabsorption is decreased, which raises the excretion of glucose in the urine (glucosuria) and lowers plasma glucose levels.

- Effects on Sodium Reabsorption and Hemodynamics

Since sodium and glucose are typically cotransported by SGLT2, its inhibition also reduces sodium reabsorption. Afferent arteriolar constriction results from the restoration of tubuloglomerular feedback brought about by the enhanced sodium transport to the distal nephron [58]. This leads to osmotic diuresis, which decreases blood pressure, as well as a little decrease in glomerular hyperfiltration, which may halt the course of diabetic nephropathy [37].

### 4.2. Pharmacodynamics Outcomes

- Glycemic Control

These substances decrease blood glucose and HbA_1_c by around 0.5–1.0% on average by encouraging glucosuria. Patients with beta-cell malfunction benefit from their action, which is independent of insulin secretion.

- Weight Loss and Blood Pressure Reduction

Weight loss is aided by the loss of glucose through urine, which is equivalent to a daily calorie loss of 200–300 kcal. Furthermore, osmotic diuresis and natriuresis cause slight drops in blood pressure, usually ranging from 3 to 6 mmHg systolic [59].

- Cardiovascular and Renal Benefits

Clinical research has shown that these medications slow the progression of renal disease and lower the risk of cardiovascular events, with empagliflozin demonstrating a significant decrease in cardiovascular mortality. It is thought that some of these renoprotective benefits are supported by the hemodynamic alterations (decrease in intraglomerular pressure).

**Figure 2:**
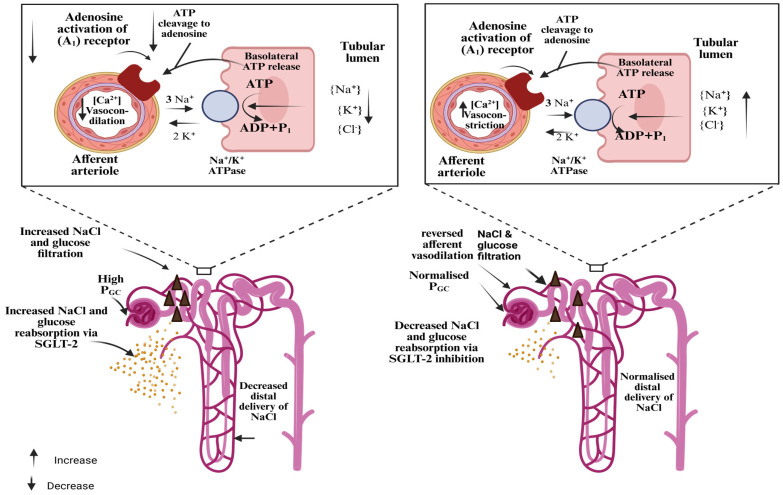
Mechanism of SGLT2i in the Treatment of CKD and DKD. Description: This figure illustrates the renal and systemic actions of SGLT2i that contribute to kidney protection in both diabetic and non-diabetic settings. By inhibiting glucose and sodium reabsorption in the proximal tubule, SGLT2i restore tubuloglomerular feedback, reduce intraglomerular pressure, and attenuate hyperfiltration. Additional benefits include improved glycemic control, reduction in body weight and blood pressure, and anti-inflammatory and anti-fibrotic effects, collectively slowing the progression of CKD and diabetes.

In conclusion, empagliflozin, dapagliflozin, and canagliflozin all work irrespective of insulin action by blocking SGLT2, which lowers blood glucose through improved urine excretion, lowers blood pressure through natriuresis, encourages weight loss, and may have protective effects on the kidneys and heart as depicted in Figure 2 [60]. Because of their complex pharmacology, SGLT2i are a useful treatment for type 2 diabetes and its aftereffects.

#### 4.2.1. Empagliflozin

A strong, competitive, and specific inhibitor of the sodium glucose transporter SGLT2, empagliflozin regulates glucose reabsorption in the beginning of the proximal tubule and the majority of the kidney’s overall glucose reabsorption. As a result, empagliflozin elevates the excretion of glucose in the urine and leads to decreases in blood glucose levels [61]. These effects are linked to pancreatic cell activity and decrease body weight in normoglycemic obese and non-obese animals regardless of a higher food intake, primarily because of a loss of adipose tissue [62,63]. Empagliflozin decreased blood pressure in diabetic individuals, and alleviated endothelial dysfunction and arterial stiffness in diabetic rats, which slowed the onset of nephropathy in diabetic animal models [61].

#### 4.2.2. Dapagliflozin

Dapagliflozin selectively inhibits SGLT2 in the early proximal tubule, reducing glucose and sodium reabsorption independently of insulin, making it effective even in patients with impaired β-cell function [62]. Beyond glycemic control, it promotes natriuresis, restoring tubuloglomerular feedback, and reducing hyperfiltration and intraglomerular pressure—key contributors to kidney damage in CKD [63]. Clinical trials have shown that dapagliflozin significantly lowers the risk of eGFR decline (≥50%), progression to end-stage kidney disease (ESKD), and cardiorenal mortality, regardless of diabetes status [64].

While its glucosuric effect can increase the risk of urinary tract and genital infections, dapagliflozin is generally well tolerated in CKD patients. Its insulin-independent action, combined with strong renal and cardiovascular benefits, supports its growing use as a key therapeutic option in CKD management, for both diabetic and non-diabetic populations [62,64].

#### 4.2.3. Canagliflozin

Canagliflozin lowers plasma glucose levels by blocking SGLT2 (irrespective of insulin) which decreases glucose reabsorption and increases glucosuria. Canagliflozin’s natriuretic action (via decreased sodium reabsorption) lowers intraglomerular pressure in addition to lowering hyperglycemia. An important factor in the development of CKD is glomerular hyperfiltration, which is reduced by this restoration of tubuloglomerular feedback [65]. The renoprotective advantages of canagliflozin in CKD patients are thought to be caused by these hemodynamic alterations.

In individuals with type 2 diabetes and CKD, canagliflozin has been demonstrated in clinical trials like the CREDENCE research to lower the risk of unfavorable kidney outcomes, such as a persistent fall in eGFR, progression to end-stage kidney disease and renal death [65]. Canagliflozin’s most common adverse effects include urinary tract infections and vaginal mycotic infections, both of which are linked to elevated urine glucose levels [66]. Potential hazards include bone fractures and, in certain assessments, an increased likelihood of lower limb amputations. An overview of different SGLT2i is depicted in Table 1.

### 4.3. Clinical Trials and Outcomes

The renoprotective and cardiovascular advantages of SGLT2i in patients with type 2 diabetes mellitus (T2DM) and DKD have been solidly demonstrated by a number of seminal randomized controlled studies (RCTs) conducted over the last ten years, as shown in Table 2. These medications cause glucosuria, which lowers blood glucose levels by inhibiting SGLT2 in the kidney’s proximal tubule. Crucially, their advantages go beyond glucose management; they also aid in lowering cardiovascular events and slowing the progression of DKD [66].

#### 4.3.1. CREDENCE Trial (Canagliflozin)

The effectiveness and safety of incorporating canagliflozin versus a placebo into standard care for patients with CKD and T2DM has been assessed in the CREDENCE trial (Evaluation of the Effects of Canagliflozin on Renal and Cardiovascular Outcomes in Participants with Diabetic Nephropathy, i.e., T2DM patients with DKD (eGFR 30–<90 mL/min/1.73 m^2^; severe albuminuria) [58]. When compared to a placebo, canagliflozin significantly decreased either the composite endpoint of renal death, serum creatinine doubling or end-stage kidney disease (ESKD). The results showed that, in patients with established DKD, SGLT2 inhibition can decrease the progression of renal disease [52,64].

#### 4.3.2. DAPA-CKD Trial (Dapagliflozin)

In a new trial, patients with albuminuria (eGFR 25–<75 mL/min/1.73 m^2^) and chronic renal disease (with or without type 2 diabetes) were randomly assigned to receive dapagliflozin (with or without saxagliptin) or a placebo. This study assessed the number of patients who reached a 30% reduction in UACR over the trial period, as well as the percentage change in UACR between dapagliflozin 10 mg + saxagliptin 2.5 mg and placebo and between dapagliflozin 10 mg and placebo during a 24-week period [64]. Comparing dapagliflozin to a placebo, the results showed that the former reduced the probability of an integrated renal endpoint (≥50% drop in eGFR, progression to ESKD, or renal/cardiovascular death). Even in those with non-diabetic CKD, dapagliflozin exhibits strong renoprotective effects, extending the potential utility of SGLT2i in kidney disease [52,58].

#### 4.3.3. EMPA-KIDNEY Trial (Empagliflozin)

The EMPEROR-Preserved and EMPEROR-Reduced trials are evaluating the safety and efficacy of empagliflozin in patients with heart failure, either with preserved or reduced ejection fraction, as an add-on to standard therapy. Meanwhile, the EMPA-KIDNEY trial is assessing empagliflozin’s impact on kidney disease progression and cardiovascular death in patients with CKD, regardless of diabetes status and down to an eGFR of 20 mL/min/1.73 m^2^ [67]. These studies build upon the landmark EMPA-REG OUTCOME trial, which showed that empagliflozin significantly reduced the risk of cardiovascular death and renal disease progression. Collectively, these trials reinforce empagliflozin’s potential as a versatile agent in managing both cardiorenal complications in diabetic and non-diabetic populations [68].

#### 4.3.4. Real-World Evidence

The RCT results have been corroborated by observational studies (e.g., CVD-REAL 3 studies from the Japan CKD Database) which show that starting SGLT2i is linked to a slower drop in eGFR and a decreased risk of ESKD in standard clinical practice. According to subgroup analysis, these advantages hold true regardless of proteinuria levels and the previous rate at which kidney function declined [69]. In addition to enhancing glycemic management, it offers significant cardiovascular and renal protection, lowering the chance of developing end-stage kidney disease and other negative consequences. These advantages seem to hold true for different patient groupings, suggesting that they could be used more widely in CKD populations with and without diabetes [70]. SGLT2i are crucial for reducing kidney and cardiovascular hazards in this high-risk patient population. The key findings of major randomized clinical trials are summarized in Table 2.

### 4.4. Meta-Analyses of SGLT2i

SGLT2i have been shown to have strong renoprotective effects in individuals with DKD in meta-analyses that have combined data from several randomized trials [71].

#### Pooled Effects on Renal Endpoints (Reduction in Composite Renal Endpoints)

According to pooled investigations, SGLT2i lower the risk of a composite renal outcome by about 30–40% when compared to a placebo. This composite renal outcome is usually defined as a prolonged fall in estimated glomerular filtration rate (eGFR), progression to end-stage kidney disease (ESKD), or renal death. For example, a well-known meta-analysis found that trials using drugs like canagliflozin, dapagliflozin, and empagliflozin had a hazard ratio (HR) for renal outcomes of approximately 0.66 [69,70,71].

- Consistency across Agents and Subgroups

Regardless of beginning kidney function or baseline albuminuria levels, the renoprotective effects of the various SGLT2i are consistent. This implies that these medications slow down the course of DKD in a wide range of patients, including those with lower initial eGFR [65].

- Mechanistic Contributions

Along with potential direct anti-inflammatory and anti-fibrotic effects on the kidney, the advantages are ascribed to hemodynamic changes, including decreased intraglomerular pressure through natriuresis as well as better glycemic management [71]. All things considered, these meta-analyses support the use of SGLT2i as a crucial part of DKD management in clinical practice and validate that they are successful in slowing the progression of kidney disease in diabetic patients. They also support the results of individual landmark trials, such as CREDENCE and DAPA-CKD [58,64,65,66].

Zelniker et al.’s (2019) meta-analysis showed that, in comparison to a placebo, SGLT2i significantly lower the risk of composite renal endpoints, such as doubling of serum creatinine, renal death, or progression to end-stage kidney disease and offered solid proof of SGLT2is’ renoprotective benefits in diabetic renal disease [66].

Heerspink et al.’s (2020), meta-analysis demonstrated the dual benefits of SGLT2i, highlighting their role in comprehensive diabetes care by confirming that they significantly reduce the progression of kidney disease and cardiovascular events in addition to improving glycemic control [58]. Neuen et al.’s (2019) meta-analysis supports the argument for SGLT2i as crucial tools in slowing the progression of DKD and was reinforced by pooled data from several trials, which showed that SGLT2 lower albuminuria and halt the decline in estimated glomerular filtration rate (eGFR) [52].

Jamil et al. (2022) [67] implicated that patients on SGLT2i had a significantly decreased risk of MACE and all-cause mortality. Furthermore, the SGLT2 inhibitor group showed a much higher change in Hb1AC. The SGLT2 inhibitor group had a considerably decreased incidence of acute renal damage, major adverse events, and hyperkalemia in terms of safety outcomes. In individuals with diabetes and CKD, the SGLT2i dramatically reduced the incidence of severe cardiovascular events and all-cause mortality. Additionally, SGLT2i work well to lower patients’ Hb1Ac levels [67].

In Brendon et al.’s (2019) study [52], SGLT2i significantly decreased the probability of dialysis, kidney disease-related death, or transplantation. Additionally, SGLT2i consistently improved acute renal damage and end-stage kidney disease across investigations. There was distinct, unambiguous evidence of benefit for all eGFR subgroups, including those with a baseline eGFR of 30–45 mL/min per 1.73 m^2^. Additionally, renoprotection was constant across studies, regardless of RAS blockade use and baseline albuminuria.

Ning Li et al.’s (2020) [52] recent trials were the first to show how sodium–glucose cotransporter-2 (SGLT2) inhibitors affected renal outcomes in patients with CKD. Patients with eGFR < 60 mL/min/1.73 m^2^ and those with UACR > 300 mg/g had a 30% and 43% lower risk of the major renal outcome, respectively, while using SGLT2i. Patients with CKD who also had type 2 diabetes showed a similar effect [52].

However, SGLT2 reduced the risk of major renal outcomes by 46% in individuals with macroalbuminuria (defined as UACR > 300 mg/g) and atherosclerotic cardiovascular disease. In CKD patients with heart failure, SGLT2i did not significantly lower the risk of serious renal outcomes. In patients with CKD, SGLT2i dramatically decreased the probability of the primary outcome. However, the renal protective effect varies for patients with diverse characteristics and underlying conditions [66].

### 4.5. Safety and Risk

Two primary safety signals are regularly reported by meta-analyses that have assessed the safety of SGLT2i in a variety of patient populations, including those with kidney disease [69].

#### 4.5.1. Euglycemic Diabetic Ketoacidosis (DKA)

Although SGLT2 inhibitor use has been linked in multiple trials to a higher relative risk of euglycemic DKA, the absolute risk is still minimal. DKA is uncommon in people with type 2 diabetes (and consequently in many with diabetic renal impairment), according to meta-analyses that include data from randomized studies [72]. Instead of being a direct pharmacological consequence of the drug itself, the majority of occurrences happen under particular triggering factors (such as severe illness, low insulin doses, or perioperative states).

#### 4.5.2. Genital Infections

When compared to a placebo, the combined data consistently demonstrate that SGLT2i are linked to an increased incidence of genital mycotic infections. Usually mild to moderate in severity, these infections—which are most frequently caused by Candida species—respond well to standard antifungal treatment and infrequently result in treatment cessation [73].

#### 4.5.3. Pooled Effects

Although SGLT2i considerably raise the risk of genital infections, the absolute number of occurrences is low and controllable with the right therapy, according to meta-analyses that aggregate data from several RCTs, including those that concentrate on patients with DKD. Despite having a higher relative risk, euglycemic DKA is still uncommon overall, especially in people with type 2 diabetes and significant CKD [74].

### 4.6. Clinical Implications

The necessity for clinicians to advise patients on the early indicators of DKA and genital infections is highlighted by these safety findings. The significant renal and cardiovascular advantages of SGLT2i can be outweighed by preventive measures, such as making sure you drink enough water, carefully adjusting your insulin or other diabetic medications during high-risk times and treating any infections as soon as they appear [75]. While SGLT2i do have certain risks, such as a higher risk of genital mycotic infections and a low but present risk of euglycemic DKA, the overall meta-analytic evidence suggests that these risks are generally outweighed by the positive effects of lowering the progression of DKD and cardiovascular events when patients are carefully chosen and closely monitored [76].

## 5. GLP-1 RAs in Kidney Disease

Oral glucose triggers a greater insulin response than intravenous glucose due to the incretin effect, primarily mediated by GLP-1 and GIP, two gut-derived hormones. GLP-1 enhances insulin and somatostatin secretion by binding to its receptor (GLP-1R) on pancreatic β and δ cells [77]. Somatostatin, in turn, suppresses glucagon release from α cells via somatostatin receptor 2, contributing to improved glycemic control. Beyond the pancreas, GLP-1 boosts insulin sensitivity and promotes weight loss by activating hypothalamic centers involved in appetite and satiety, forming part of the gut–brain axis [78]. During meals, GLP-1 also stimulates vagal sensory fibers via GLP-1R in the portal vein, enhancing metabolic signaling.

**Figure 3:**
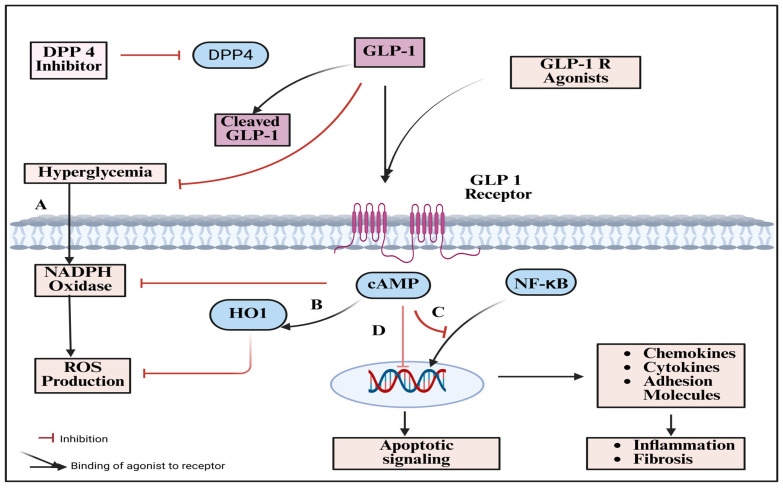
Mechanistic Insights into Incretin Signaling Pathways in Renal Cells. Description: (A,B) GLP-1 RAs activate the G protein-coupled GLP-1R, directly reducing reactive oxygen species (ROS) production and upregulating heme oxygenase-1 (HO-1), a key antioxidant enzyme. DPP-4 inhibitors prolong endogenous GLP-1 activity by preventing its enzymatic degradation, indirectly enhancing GLP-1R signaling. (C) GLP-1R activation inhibits NF-κB p65 binding to proinflammatory gene promoters, thereby reducing the expression of inflammatory and fibrotic mediators. (D) DPP-4 inhibitors lower the BAX/BCL-2 and BIM/BCL-2 ratios, suggesting attenuation of apoptosis and enhanced cellular survival.

Moreover, GLP-1R activation reduces lipid accumulation in white adipose tissue and increases energy expenditure in brown adipose tissue via sympathetic nervous system pathways, independent of physical activity [79,80]. However, DPP-4 enzymes rapidly degrade GLP-1 into an inactive form within 1–2 min of its release, limiting its natural duration of action as depicted in Figure 3.

### 5.1. Pharmacological Properties of GLP-1RAs

GLP-1RAs mimic endogenous GLP-1 by activating GLP-1R without being degraded by DPP-4 or interfering with GIP, thereby enhancing both local and systemic effects. Their glucose-dependent insulinotropic action accounts for the low risk of hypoglycemia, even when used alone or in combination with metformin, pioglitazone, or basal insulin [79].

**Figure 4:**
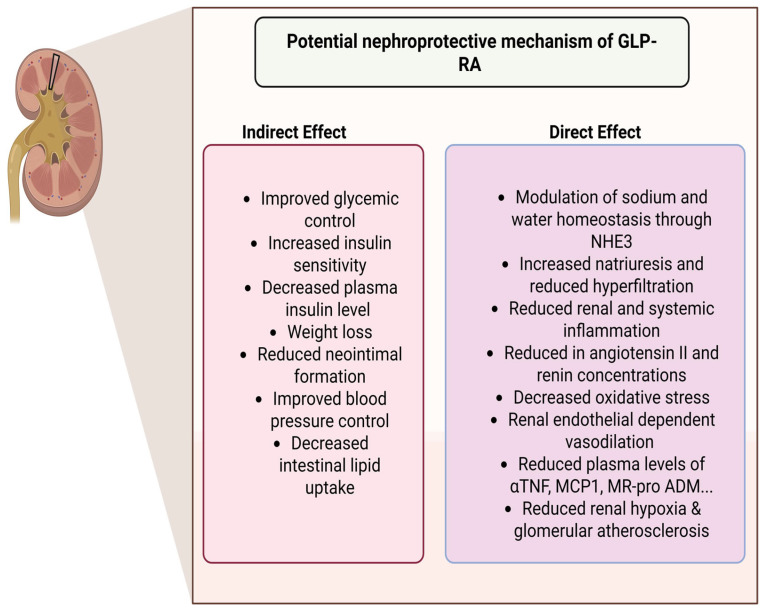
Potential nephroprotective mechanisms of GLP-1 RAs in the Treatment of CKD and DKD. Description: This figure depicts the multifaceted actions of GLP-1 RAs that contribute to renal protection in CKD and DKD. These include improved glycemic control, weight reduction, and blood pressure lowering. Additionally, GLP-1 agonists exert direct renal effects through anti-inflammatory, antioxidative, and anti-fibrotic pathways, reduction of albuminuria, and modulation of renal hemodynamics. These combined effects help slow the progression of kidney damage and support their emerging role in cardiorenal risk reduction.

GLP-1RAs are categorized as incretin mimetics, including Lixisenatide (Lyxumia^®^), Exenatide (Byetta^®^), and Exenatide LAR (Bydureon^®^). Although resistant to DPP-4 degradation, these agents share ~52% homology with native GLP-1, making them immunogenic with the potential to induce neutralizing antibodies [77]. Except for LAR formulations, they are considered short-acting due to transient plasma peaks and primarily delay gastric emptying, effectively reducing postprandial glucose spikes. These agents are cleared via glomerular filtration, tubular reabsorption, and proteolysis, and should be avoided in patients with eGFR < 30 mL/min/1.73 m^2^ due to impaired renal clearance [80,81]. The potential nephroprotective mechanism of GLP-1 RAs is depicted in Figure 4.

### 5.2. Human GLP-1 Analogs

Injectable semaglutide (Ozempic^®^), oral semaglutide (Rybelsus^®^), dulaglutide (Trulicity^®^), ligarglutide (Victoza^®^, Saxenda^®^), and albiglutide (Eperzan^®^) are human GLP-1 analogs. They are also referred to as “long-acting” because, once the steady state is achieved, they sustain high blood concentrations, enabling ongoing GLP-1R activation with relatively little variation between doses [81]. These analogs have a long half-life and are not eliminated by the kidneys because of certain molecular properties, such as the covalent link with albumin (albiglutide), the Fc region of human immunoglobulin (Ig) G4 (dulaglutide), or particular fatty acids (liraglutide). Therefore, even at eGFR values as low as 15 mL/min/1.73 m^2^, human GLP-1 analogs could be utilized safely.

These medications are catabolized in target tissues similarly to big proteins, with no primary elimination pathway particular to an organ [21]. Human GLP-1 analogs have demonstrated more efficacy on cardiovascular mortality and morbidity than short-acting GLP-1RAs. They also cause a more significant reduction in HbA1c and fasting blood sugar, as well as a decrease in the occurrence of side effects such nausea and vomiting. The advantages of human GLP-1 analogs in CKD and DKD are as follows:

- Lower Immunogenicity—Reduced risk of immune system reactions due to structural similarity to native GLP-1.
- Extended Half-Life—Enhanced stability leads to prolonged activity and less frequent dosing.
- Improved Glycemic Control—More sustained glucose-lowering effects with reduced fluctuations.
- Greater Cardiovascular Benefits—Proven to lower cardiovascular risk in high-risk patients.
- Enhanced Renal Protection—Potential to reduce kidney inflammation and slow CKD progression.
- Better Tolerability—Lower likelihood of antibody formation compared to non-human GLP-1 RAs.

Oral semaglutide (Rybelsus^®^)—the first once-daily, ingestible GLP-1RA—can be used even in advanced renal impairment (eGFR ≥ 15 mL/min/1.73 m^2^) because its pharmacokinetics remain largely unchanged. GLP-1RAs mitigate glomerular hyperfiltration by phosphorylating and inhibiting the proximal-tubular Na^+^/H^+^ exchanger 3, thereby promoting natriuresis and diuresis [21,81]. They further slow DKD by boosting intraglomerular nitric-oxide availability, suppressing endothelial profibrotic signals, and curbing mesangial expansion. Additional renoprotection arises from their anti-atherogenic actions—dampening chylomicron synthesis, lowering LDL-C and triglycerides, and fine-tuning renal-cell mitochondrial function. At the molecular level, GLP-1RAs elevate cAMP/PKA activity to blunt oxidative stress, while down-regulating NADPH oxidase, AGE-receptor expression, and NF-κB signaling [82]. Collectively, these mechanisms explain the broad kidney benefits of oral semaglutide and other GLP-1RAs across the CKD spectrum. Different GLP-1 agonists are depicted in Table 3.

### 5.3. Clinical Outcomes

#### 5.3.1. LEADER Trial

In the LEADER trial, diabetic patients with recognized cardiovascular conditions or significant risk were randomized to either the liraglutide or placebo group [83]. The liraglutide group experienced a mean decrease of 2.3 kg in body weight and 0.4% in HbA1c after an average follow-up of 3.8 years [84]. According to the primary endpoint, the liraglutide group experienced a lower incidence of MACEs (13%) than the placebo group (14.9%). The recurrence of nephropathy was 22% lower in the liraglutide group compared to the placebo group and was particularly beneficial for macroalbuminuria. This effect was most noticeable in subgroups of individuals with moderate (eGFR 30–59 mL/min/1.73 m^2^) or severe (eGFR < 30 mL/min/1.73 m^2^) CKD. The eGFR fall was lower in the liraglutide group.

#### 5.3.2. SCALE Study

Liraglutide’s effectiveness in controlling body weight in overweight or obese diabetes individuals was assessed in the SCALE study. Patients were randomized to receive either a placebo or 3 mg or 1.8 mg of liraglutide. Both liraglutide arms experienced a significant decrease in weight at the end of the 56-week research period; however, their urine albumin/creatinine ratios (UACR) were lower than those of the placebo group [85]. Liraglutide turned out to be unsuccessful in improving renal functions in a sample of 279 diabetic patients with intermediate CKD (eGFR 30–59 mL/min/1.73 m^2^), in contrast to the LEADER and SCALE trials [86].

#### 5.3.3. SUSTAIN

Ten randomized controlled trials in the “SUSTAIN” series are designed to assess the impact of weekly administration of subcutaneous semaglutide on glycemic management in individuals with type 2 diabetes. The most widely prescribed medications for type 2 diabetes (T2DM) (sitagliptin, exenatide, insulin glargine, dulaglutide, canagliflozin, and liraglutide) were compared with semaglutide administered either alone or combined with insulin, metformin, sulfonylurea, and/or insulin [87]. Semaglutide treatment does not raise the chance of adverse renal events when compared to other antidiabetic treatments [85]. After 2.1 years of follow-up, the semaglutide arm outperformed the placebo for MACEs (6.6% versus 8.9%), glycemic control (mean HbA1c −1.1% versus −1.4%), weight loss (−3.6 vs. −4.9 kg), and the onset or worsening of nephropathy (3.8% versus 6.1%).

#### 5.3.4. ELIXA Research

The purpose of the randomized, double-blind, parallel-group ELIXA research was to examine the effects of lixisenatide and a placebo on cardiovascular risk in individuals with diabetes who had recently experienced an episode of acute coronary syndrome [86]. The incidence of MACEs was one of the key composite endpoints that were evaluated for superiority and non-inferiority. According to the study, lixisenatide’s cardiovascular safety is no worse or better than that of a placebo. Lixisenatide avoids the development of macroalbuminuria in participants who were initially normoalbuminuric (−1.69%) and lowers the UACR variance in both microalbuminuric (−21%) and macroalbuminuric (−39%) patients at baseline [88].

#### 5.3.5. EXSCEL Trial

In the EXSCEL trial, diabetic subjects were randomly assigned to receive exenatide LAR at a dose of 2 mg weekly versus placebo for a 3.2-year observation period. The findings demonstrated that exenatide is not superior in terms of efficacy in averting MACEs and is comparable to placebo in terms of safety across every group of patients with CKD of varying degrees [89]. Subsequent analysis of the data modified for baseline demographics and comorbidities showed an important improvement in the renal composite outcome, mainly explained by a lower incidence of macroalbuminuria, even though exenatide did not significantly enhance the decline in eGFR, the incidence of ESRD, or renal-related death in the EXSCEL trial [81,89].

#### 5.3.6. AWARD-7

In the AWARD-7 study, diabetic patients with CKD stages G3 and G4 were recruited and randomly assigned. HbA1c changes at 26 weeks were the main outcome [87]. The results showed that dulaglutide safely and efficiently improves glycemic control in diabetic patients with advanced renal disease after 52 weeks of monitoring. Although there were no statistically significant variations in the reduction of UACR, dulaglutide was more effective than insulin glargine in reducing the decline in renal function in terms of secondary endpoints. These findings obliquely support the idea that weight reduction in GLP-1RA-treated patients is due to a decrease in fat mass rather than muscle mass [90].

#### 5.3.7. REWIND Trial

In the multicenter, double-blind, placebo-controlled REWIND trial, diabetic individuals with cardiovascular medical conditions or a history of cardiovascular events were randomized. The prevalence of MACEs using an intention-to-treat strategy was the main result [91]. The percentages of eGFR fall ≥ 30% and the requirement for dialysis revealed a trend that was nearly identical in both groups, despite the fact that the incidence of macroalbuminuria was 8.9% compared to 11.3% in the placebo group [86]. The meta-analysis GLP-1RAs do not just lower the incidence of MACEs, heart failure hospitalization, and all-cause mortality, but they also improve the composite renal consequence in terms of eGFR decline over time [85,91].

#### 5.3.8. FLOW Trial

The impact of semaglutide vs. placebo on the development of renal impairment in participants with type 2 diabetes and CKD (FLOW trial) was initiated in 2019 in order to assess semaglutide’s efficacy to lower the incidence of the composite primary endpoint (eGFR decline ≥ 50% from baseline, requirement for dialysis, death from renal causes, and death from cardiovascular disease) in comparison to a placebo [92]. The study found the true function of GLP-1RAs as medications that can prevent the progression of DKD in individuals with type 2 diabetes, as depicted in Table 4.

### 5.4. Meta-Analyses of GLP-1 RA

Seven trials—ELIXA (lixisenatide), LEADER (liraglutide), SUSTAIN-6 (semaglutide), EXSCEL (exenatide), Harmony Outcomes (albiglutide), REWIND (dulaglutide), and PIONEER 6 (oral semaglutide)—with participants from 27 publications were evaluated. MACE decreased by 12% overall with GLP-1 receptor agonist treatment. GLP-1 receptor agonist treatment decreased hospitalization for heart failure by 9%, all-cause mortality by 12% and broad composite kidney results (development of new-onset macroalbuminuria, decline in estimated glomerular filtration rate, progression to end-stage kidney disease, or death attributable to kidney causes) by 17%, primarily as a result of decreased urinary albumin excretion [93]. GLP-1 RAs regularly result in mild decreases in albuminuria in individuals with type 2 diabetes and diabetic renal disease. As a result, even though GLP-1 RAs have renoprotective effects beyond glycemic management, it is unclear whether they will improve hard renal outcomes, which emphasizes the need for renal outcome trials [94].

Krisanapan et al. (2024) [95] performed a meta-analysis to assess GLP-1RAs’ effectiveness and safety in this population, including observational studies and clinical trials that discussed the safety or effectiveness of GLP-1RAs in adult KTRs. Weight, cardiovascular outcomes, adverse events, glycemic and metabolic markers, and kidney graft performance were assessed. Weight, body mass index, and total daily insulin dosage all sharply declined [95]. The most common side effects were nausea and vomiting (17.6%), diarrhea (7.6%), and soreness at the injection site (5.4%). Without changing tacrolimus levels, GLP-1RAs help KTRs lose weight, improve glycemic management, and lessen proteinuria. The most common adverse effects are gastrointestinal problems.

Mohamed et al. (2024), in their systematic review, chose eleven studies, all of which offered enough information to support the renoprotective effect of GLP-1 receptor agonist [96]. All things considered, this meta-analysis supports the use of GLP-1 RAs as a therapeutic option to preserve renal function in patients with type 2 diabetes, especially those who already have or are at high risk of developing DKD.

In Liu et al.’s (2019) study [39], GLP-1 RAs were found to have minimal impact on hard endpoints, such as end-stage renal disease, but dramatically decrease the course of albuminuria and exhibit trends toward a slower drop in eGFR. The results suggest that GLP-1 RAs have a function in renoprotection, specifically in preventing microvascular damage, in addition to their well-established advantages in weight loss and glycemic management [39].

In Mann et al.’s (2017) study [90], the results of several RCTs were combined to show that albuminuria levels were significantly decreased by GLP-1 RAs medication. Even while the eGFR drop improvement was not as strong as it was with SGLT2i, the data points to a generally positive renal effect [90]. Even if the amount of protection against harsh renal outcomes is still limited, our data show that GLP-1 RAs have a beneficial effect on surrogate renal indicators and may be taken into consideration in the management of DKD.

Sattar et al.’s (2021) [91] meta-analysis looked at secondary renal endpoints from big cardiovascular outcome trials; however, its primary goal was to evaluate cardiovascular outcomes. It was found that GLP-1 RAs have the ability to halt the decrease in kidney function and was linked to a lower incidence of macroalbuminuria. The research shows that GLP-1 RAs regularly show advantages that suggest an additional layer of renoprotection in patients with type 2 diabetes, even when renal outcomes are secondary endpoints [91].

Although their impact on more definitive kidney outcomes is less pronounced than that of SGLT2i, Allegretti et al.’s (2019) review and meta-analysis combined data from both cardiovascular outcome studies and dedicated renal trials to conclude that GLP-1 improves surrogate markers like albuminuria and may favorably affect the eGFR slope [97]. The study backs up the idea that GLP-1 RAs are a good choice, particularly for individuals with early renal involvement, because their renoprotective effects are mainly caused by enhancements in metabolic regulation and anti-inflammatory mechanisms.

### 5.5. GLP-1/GIP Agonist

The primary physiological function of the incretin hormones glucagon-like peptide-1 (GLP-1) and glucose-dependent insulinotropic polypeptide (GIP) is to increase insulin secretion following their nutrient-induced secretion from the stomach. Maintaining a normal glucose tolerance requires a healthy entero-insular (gut-endocrine pancreas) axis. The incretin effect, which is a higher insulin secretory response to oral glucose delivery as opposed to “isoglycemic” intravenous glucose administration, because of the release and activity of incretin hormones, serves as an example of this. The effects of GIP and GLP-1 on insulin secretion are cumulative. The discovery that GIP/GLP-1 receptor co-agonists, such as tirzepatide, are more effective than selective GLP-1 RAs in terms of body weight and glycemic control has rekindled interest in GIP, which was previously believed to have no therapeutic potential [90,97].

#### 5.5.1. Tirzepatide

Tirzepatide, a dual GLP-1/GIP agonist, is a new treatment option for those with type 2 diabetes and DKD. Despite the fact that tirzepatide was first created to help with weight loss and glycemic control, new evaluations of the SURPASS trials have revealed encouraging results for renal outcomes. In particular, tirzepatide has been linked to slower eGFR decline and decreases in albuminuria indicating possible renoprotective actions in addition to its metabolic advantages. Whether tirzepatide alone or in conjunction with other medications, such as SGLT2i, can further improve kidney outcomes in comparison to monotherapy is now being investigated in ongoing combination therapy trials [98]. The rationale behind these studies depends on the complementary mechanisms of action: SGLT2i lower intraglomerular pressure and offer strong cardiovascular and renal protection, while tirzepatide enhances glycemic control and has anti-inflammatory effects through dual incretin receptor activation. These actions could work in concert to decrease the evolution of diabetic nephropathy [99].

#### 5.5.2. Preclinical Studies

Tirzepatide has been shown to enhance glycemic control, lower albuminuria, and lessen renal inflammation and fibrosis in animal models of diabetic nephropathy. According to these preclinical results, dual incretin receptor activation may slow the progression of kidney injury by lowering inflammation and oxidative stress. According to preclinical evidence, tirzepatide may directly affect renal tissue, possibly improving tubular repair and lowering glomerular hyperfiltration, in addition to improving systemic metabolic parameters [100].

#### 5.5.3. Clinical Evidence

The main goals of the SURPASS clinical trial series were weight loss and glycemic management in individuals with type 2 diabetes. Secondary analyses, however, have revealed encouraging kidney signs:

- Reduced albuminuria levels, which may indicate a slowing of diabetic kidney damage.
- Trends toward a slower decline in eGFR in patients with pre-existing kidney impairment.
- Continuous analyses are assessing whether the enhancements in metabolic regulation result in long-term renoprotection, even though strong, specialized kidney outcome trials for tirzepatide are still being developed.

#### 5.5.4. Post-Clinical Evidence

Since tirzepatide has just recently hit the market, empirical data pertaining to DKD is starting to surface. Confirming the persistence of renoprotective effects and comprehending its influence on hard kidney endpoints, such as the rate of decline in eGFR and the progression to end-stage kidney disorders, would require post-marketing observation. The long-term relationship between GIP and GLP-1 receptor signaling is one area of study [96]. The unresolved questions will presumably be clarified by the introduction of GLP-1 receptor antagonists (exendin (9–39]) and, more recently, GIP receptor agonists (GIP (3–30] NH_2_), as well as, ideally, longer-acting GIP receptor agonists for human usage.

## 6. Comparative Analysis: SGLT2i vs. GLP-1 RA

Both drug classes improve outcomes in DKD, but they do so via complementary pathways. SGLT2i tend to have a stronger and more consistent effect on hard renal outcomes, whereas GLP-1 receptor agonists may offer additional benefits in reducing albuminuria and systemic inflammation [90]. This distinction can help clinicians tailor therapy based on a patient’s overall risk profile, utilizing SGLT2i when hemodynamic modulation is paramount and considering GLP-1 receptor agonists for their broader metabolic and anti-inflammatory advantages, as depicted in Table 5.

The key outcomes from pivotal trials investigating GLP-1 RAs and SGLT2i in CKD and DKD populations are depicted in Table 6. While GLP-1 RAs primarily demonstrate benefits in reducing albuminuria and slowing eGFR decline, SGLT2i provide robust protection against renal disease progression, ESKD, and renal-related mortality. The combined evidence supports their complementary roles in modern cardiorenal metabolic management.

### 6.1. Combination Therapy Witnessing Emerging Evidence

A substantial decrease in cardiovascular and renal events was shown in the SCORED study, which assessed the dual SGLT1/2 inhibitor sotagliflozin in patients with CKD and DKD. SCORED highlights the potential advantages of SGLT2i combining with GLP-1 RAs could further enhance outcomes in high-risk individuals. When compared to monotherapy, recent real-world studies and network meta-analyses show that the combination can result in greater reductions in body weight and HbA1c, with possible extra advantages on blood pressure and lipid profiles [101].

The general safety profile is still acceptable, even though combination therapy may raise the incidence of particular adverse events (such as gastrointestinal side effects from GLP-1 RAs and a modest increase in urinary tract infections with some SGLT2i). This is especially true when patients are carefully chosen, and both medications are titrated correctly. The combination of SGLT2i and GLP-1 RAs seems encouraging for individuals with type 2 diabetes that are at high risk for cardiovascular and renal problems, such as those who have CKD. According to new results from studies like SCORED, the improved effectiveness in glycemic management and weight loss may result in further decreases in significant adverse cardiovascular events and a slowing of the progression of kidney disease.

**Figure 5:**
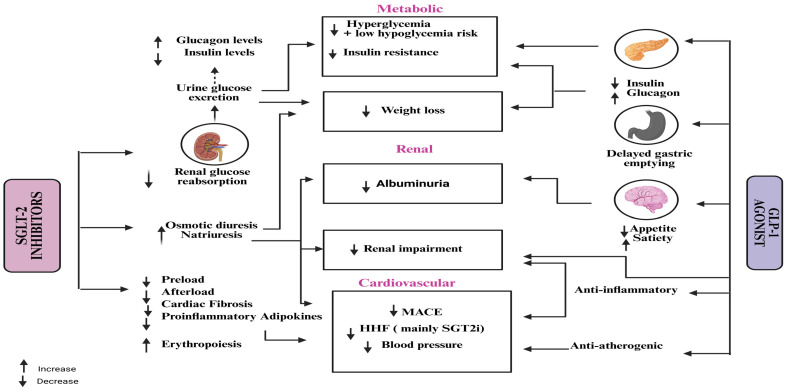
An integrated diagram that shows the complementary mechanisms when SGLT2i and GLP-1 receptor agonists are used together, highlighting potential synergistic effects on metabolic and renal endpoints. Description: SGLT2i reduce intraglomerular pressure, natriuresis, and hyperfiltration, while GLP-1 RAs enhance glycemic control, promote weight loss, lower blood pressure, and reduce inflammation. Together, they target complementary pathways, resulting in improved metabolic balance, reduced albuminuria, and slower renal disease progression.

To completely determine the long-term advantages and safety profile of this combination method, more focused “head-to-head” and combination trials are necessary as depicted in Figure 5 [90,96,100,101].

### 6.2. Patient Stratification and Comorbidity-Driven Selection

Phenotype-guided therapy, another name for patient stratification, attempts to customize treatment options according to each patient’s unique clinical traits and comorbidities. In this regard, there is strong evidence to support giving SGLT2i priority for patients who have simultaneous heart failure or a rapid loss in kidney function (e.g., an eGFR drop of more than 5 mL/min/1.73 m^2^ annually). Clinical results are improved when therapy is tailored to the patient’s characteristics [21]. There is proof that the renoprotective and hemodynamic benefits of SGLT2i are substantial for patients whose eGFR drop is greater than 5 mL/min/year. By decreasing the progression of kidney disease, these medicines lower intraglomerular pressure by natriuresis and osmotic diuresis.

SGLT2i are a particularly appealing choice for heart failure patients, irrespective of their glucose level. By using a comorbidity-driven selection approach, physicians can give SGLT2i priority for patients whose phenotype has been characterized by heart failure or accelerated loss of kidney function predicts more favorable outcomes from these medications. This specialized strategy can guide safe and economical diabetes control while improving cardiovascular and renal outcomes.

GLP-1 receptor agonists are becoming a more appealing therapy alternative for patients whose phenotype is influenced by obesity-predominant CKD, post-transplant diabetes, or significant macroalbuminuria. The strong weight-loss, anti-inflammatory, and metabolic benefits of GLP-1 RAs are frequently advantageous to these patients [102]. GLP-1 RAs provide substantial weight loss and enhance insulin sensitivity in patients with CKD, where obesity is a primary cause of metabolic stress and inflammation. Their effects on stomach emptying and satiety aid in calorie restriction, which is especially advantageous for obese people and may also indirectly lower albuminuria.

After a kidney transplant, managing diabetes presents special difficulties since patients frequently need glycemic management to prevent further weight gain and lower their risk of cardiovascular disease [90,103]. Although the direct impact on objective renal endpoints is not as strong as with SGLT2i, lowering weight and systemic inflammation may assist with improving renal outcomes in patients with macroalbuminuria.

### 6.3. Comorbidity Matrix

Both SGLT2i and GLP-1 receptor agonists have shown comparable decreases in major adverse cardiovascular events (MACE) in patients with established ASCVD. However, due to their strong advantages in lowering heart failure hospitalizations and cardiovascular mortality, the evidence shifts in favor of SGLT2i when heart failure is also present. This comorbidity-driven strategy, also known as a “comorbidity matrix” or phenotype-guided therapy, proposes that SGLT2i need to be the first line of treatment for patients with ASCVD and concomitant heart failure. From 9.2% of patients in 2018 to 27.1% in 2022, the prevalence of GLP-1 RA and/or SGLT2i use rises, with qualifying yearly patient numbers ranging from 279,474 to 348,997 [104]. During this time, SGLT2i-only use increased from 2.8% to 12.2% while GLP-1 RA-only use increased from 5.2% to 9.9%.

GLP-1 RA and/or SGLT2i incident use rose from 5.9% to 17.0% in the year after ASCVD diagnosis (2018–2022). This rise was from 3.6% to 7.8% for GLP-1 RA alone and from 1.8% to 7.0% for SGLT2i alone. Given the significant morbidity and mortality of ASCVD and its prevalence, the use of GLP-1 RAs/SGLT2is in patients with T2D and ASCVD has increased recently in the United states of America, but it is still not at its best [105].

- Type 2 Diabetes

Both GLP-1 receptor agonists and SGLT2i are authorized and often-used treatments for type 2 diabetes; they both provide glucose reductions, cardiovascular advantages, and, in the case of GLP-1 RAs, further weight loss.

- Type 1 Diabetes

Since off-label usage in type 1 diabetes is restricted due to safety concerns, specifically an elevated risk for euglycemic diabetic ketoacidosis, SGLT2i are not permitted for this population. However, even though GLP-1 receptor agonists are mainly prescribed for type 2 diabetes, their ability to improve metabolic parameters and weight control has led to their experimental use in a small number of type 1 patients (under close observation), suggesting a wider therapeutic window in terms of patient phenotype. Because of the advantages they offer for heart failure, SGLT2i are recommended. Both classes work well for type 2 diabetes, but because SGLT2i have serious off-label hazards, GLP-1 RAs are typically more adaptable for type 1 diabetes [78,105].

## 7. Guideline Integration and Real-World Evidence

### 7.1. KDIGO 2024 Update

SGLT2i should be utilized as the first-line adjunct to renin–angiotensin–aldosterone system (RAAS) inhibition in patients with CKD, irrespective of whether the patient has diabetes, according to the KDIGO 2024 update. This recommendation is based on solid clinical trial results as well as empirical evidence that SGLT2i can enhance cardiovascular outcomes, slow the progression of kidney disease, and minimize the risk of heart failure events—even in populations without diabetes. Clinicians can more successfully address intraglomerular hypertension and hyperfiltration, which are important factors in the progression of CKD, by combining these medications with RAAS blockers, providing a more thorough renoprotective approach [106].

These conclusions have been supported by real-world data, which demonstrate that SGLT2i, when combined with RAAS blocking, enhance outcomes for a wide range of patients, including those without diabetes. The KDIGO 2024 guidelines now suggest SGLT2i as the recommended adjuvant therapy to RAAS inhibitors for all patients with CKD, irrespective of their glucose status, in light of this research. When taken as a whole, these results highlight the paradigm shift in CKD care toward a more phenotype-driven treatment approach, with SGLT2i being essential for optimizing cardiovascular and renal endpoints [107].

GLP-1 RAs have become a useful treatment option for patients with early-stage CKD who exhibit metabolic syndrome and substantial albuminuria. Beyond glycemic control, they also provide weight loss, improvements in metabolic parameters, and a moderate reduction in albuminuria, which makes them especially helpful in CKD that is predominantly caused by obesity. GLP-1 RAs decrease albumin excretion, most likely via improving endothelial function and having anti-inflammatory effects. In early-stage CKD with metabolic syndrome and macroalbuminuria, GLP-1 RAs are particularly helpful because of their weight-loss and anti-inflammatory properties, which can further reduce renal risk.

### 7.2. Pragmatic Trial Insights

#### 7.2.1. EMPA-KIDNEY Trial; EMPA-KIDNEY vs. FLOW Trial Contrasts

This groundbreaking study showed that empagliflozin dramatically slowed the deterioration of kidney function and decreased major renal endpoints (e.g., progression to end-stage kidney disease and renal death) in a large population with CKD, including a considerable proportion of non-diabetic patients. Hemodynamic effects (such as natriuresis and decreased intraglomerular pressure) account for a major portion of its advantages, and they work regardless of diabetes status [108].

#### 7.2.2. FLOW Trial (GLP-1 RA)

The FLOW study, on the other hand, assesses the effectiveness of a GLP-1 receptor agonist (such as semaglutide), mainly in diabetic CKD. When opposed to SGLT2i, GLP-1 RAs appear to have fewer direct renoprotective advantages, particularly in non-diabetic CKD populations, despite their strong glycemic control, weight loss and small reductions in albuminuria. Furthermore, GLP-1 RAs may not adequately address the hemodynamic stress observed in advanced CKD due to their reliance on anti-inflammatory and metabolic enhancement mechanisms [109].

### 7.3. Veterans Affairs Cohort Data: 32% Lower Mortality with SGLT2i vs. 28% with GLP-1 RA

A study identified incident users of SGLT2i vs. DPP4i vs. GLP1a monotherapy among US veterans with diabetes who were treated in the Veterans Affairs (VA) healthcare system between 2004 and 2019. Using multivariable Cox models, the relationships between SGLT2i and DPP4i and GLP1a use and the risk of infection-related (primary outcome) and genitourinary infection hospitalizations (secondary outcome) in analyses stratified by CKD status, which is defined by estimated glomerular filtration rate and albuminuria, were investigated [101].

While GLP1a use showed similar risk, SGLT2i usage was linked to a decreased infection-related hospitalization risk in both the total and non-CKD groups when compared to DPP4i use of 0.74 and 0.77, respectively. However, the usage of GLP1a and SGLT2i was linked to decreased risk in the CKD cohort of 0.91 and 0.70, respectively [108]. SGLT2i usage was linked to a decreased risk of hospitalization for genitourinary infections in the general, non-CKD, and CKD cohorts, while GLP1a use demonstrated a risk that was comparable to that of DPP4i use [110].

In a weighted, variable-adjusted cohort using pairwise comparisons, GLP-1 RAs were associated with a modest but significant reduction in MACE and heart failure hospitalizations compared to DPP-4 inhibitors, with an adjusted risk difference of 3.2 events per 1000 person-years. Conversely, SGLT2i showed no significant association with reductions in MACE or heart failure when compared to DPP-4 inhibitors, suggesting that GLP-1 RAs may offer greater cardiovascular benefits in this comparative context [111].

### 7.4. Comparative Insights from Different Studies

Although randomized controlled trials (RCTs) have demonstrated the individual efficacy of SGLT2i and GLP-1 RAs in improving renal and cardiovascular outcomes, direct head-to-head comparisons within the same cohort remain limited. However, several real-world and observational studies have begun to shed light on their comparative performance in routine clinical practice.

#### 7.4.1. EMPRISE Analyses

EMPRISE focused specifically on empagliflozin versus DPP-4 inhibitors, but secondary comparisons with GLP-1 RAs were also evaluated. These studies showed that SGLT2i provided superior renal protection, especially in reducing hospitalizations for heart failure and slowing CKD progression, even in patients with existing DKD [108,109].

#### 7.4.2. GRADE Trial

The GRADE trial, while primarily designed to assess glycemic durability, provided some insights into cardiovascular and renal outcomes among multiple drug classes. Although GLP-1 RAs and SGLT2i were not directly compared, subgroup analyses suggested favorable weight and albuminuria reduction with GLP-1 RAs, and stronger hemodynamic and renal filtration benefits with SGLT2is, supporting their complementary roles [112].

#### 7.4.3. Meta-Analysis

A network meta-analysis comparing cardiovascular and renal outcomes across major trials concluded that both drug classes significantly reduce major adverse cardiovascular events (MACE) and progression of kidney disease. However, SGLT2i were more effective in reducing hospitalization for heart failure and progression to end-stage renal disease, whereas GLP-1 RAs showed slightly stronger effects on reducing stroke and non-fatal cardiovascular events [112,113].

#### 7.4.4. Real-World Comparative Studies

Observational comparative studies from registries (e.g., Scandinavian National Diabetes Registries, U.S. Optum and Medicare claims databases) have demonstrated that SGLT2i are associated with more consistent reductions in ESKD risk, while GLP-1 RAs show stronger effects on macrovascular outcomes and weight loss, particularly in obese or high-risk patients. Furthermore, SGLT2i were associated with a significantly lower risk of serious renal events (HR 0.76), while MACE rates were similar. Notably, combined use of both classes has shown additive benefits in recent retrospective analyses, though prospective confirmation is ongoing [114].

While definitive randomized controlled trials are still lacking, real-world data consistently show SGLT2i outperform GLP-1 RAs in renal outcomes, whereas GLP-1 RAs may offer additional cardiometabolic benefits, such as weight loss and improved endothelial function. These findings support a phenotype-driven or comorbidity-guided treatment approach in DKD/CKD management.

## 8. Unanswered Questions and Future Directions

### 8.1. Combination Therapy Trials

An ongoing clinical investigation called the COMBINE-KIDNEY trial compares the effects of monotherapy (using either medication alone) and combination therapy (using an SGLT2 inhibitor plus a GLP-1RAs) on the rate of eGFR decline in individuals with CKD. The goal of the trial is to determine whether the complementary mechanisms—the anti-inflammatory and metabolic benefits of GLP-1 RAs and the ability of SGLT2i to lower intraglomerular pressure and mitigate hyperfiltration—can provide a greater renoprotective effect, as indicated by a flatter (less negative) eGFR slope over time [115].

#### 8.1.1. Consideration for the Trials

SGLT2i have continuously demonstrated advantages in lowering cardiovascular events and delaying the deterioration of kidney function. Additionally, GLP-1 RAs help with weight loss, glycemic control, and inflammation reduction, all of which may indirectly safeguard renal function. When these two groups are combined, kidney function preservation may result in additive or even synergistic effects.

#### 8.1.2. Designing and Targeting of the Clinical Trial

It is probable that CKD patients, whether diabetic or not, are being recruited and categorized based on their initial albuminuria and eGFR results. The change in the eGFR slope (rate of decline), which offers a sensitive indicator of the preservation of renal function, is frequently the main endpoint. Safety profiles, changes in albuminuria, and cardiovascular outcomes are examples of secondary objectives.

#### 8.1.3. Implications

A paradigm shift in the treatment of patients at high risk for progressive renal disease may result if combined therapy shows a noticeably slower drop in eGFR than monotherapy. The results might lend credence to the inclusion of dual therapy in CKD clinical guidelines, providing a more thorough method of renoprotection. With the goal of utilizing the unique mechanisms of both drug classes to improve long-term results for patients with CKD, this trial is a part of the changing landscape of combination medicines in the nephrology sector [112,113,114,115].

### 8.2. Biomarker-Driven Personalization via Urinary EGFR Ligand Profiling for SGLT2i

SGLT2i urinary EGFR ligand profiling is becoming a viable technique for individualized treatment in DKD. In tubular cell regeneration and repair, EGFR ligands, including amphiregulin, heparin-binding EGF (HB-EGF), and epidermal growth factor (EGF), are essential. Reduced urine EGF levels have been linked to more severe tubular damage and accelerated renal dysfunction progression in DKD [113]. A baseline profile of EGFR ligands may help predict which individuals will respond better to SGLT2i because they partially exert their renoprotective effects through actions on the proximal tubule (e.g., lowering hyperfiltration and modulating tubular stress).

Higher baseline urine EGF levels, for instance, would predict a better response to SGLT2 inhibitor medication and suggest a comparatively retained tubular regenerative capability. Patients with extremely low urine EGFR ligand levels, on the other hand, may have more severe tubular damage and be less likely to benefit much from SGLT2 inhibition alone. Clinicians may be able to group patients according to the condition of their renal tissue by incorporating urinary EGFR ligand profiling into clinical practice.

This biomarker-driven strategy would help choose treatments based on each patient’s unique renal pathophysiology, possibly maximizing the usage of SGLT2i and directing choices about combination medications. All things considered, urinary EGFR ligand profiling is a step toward personalized medicine in nephrology, where molecular markers aid in long-term outcome improvement and treatment response prediction in DKDs [116].

#### Biomarker-Driven Personalization via Genomic Variations

TCF7L2 gene variants are one of the most potent genetic risk factors for type 2 diabetes, and new research indicates they may also affect how the body reacts to GLP-1 RAs. Specifically, decreased incretin impact and impaired insulin secretion have been associated with risk alleles like the T allele of the rs7903146 variation. In certain patients, this could result in a decreased therapeutic response or resistance to GLP-1 RA treatment. Genotyping TCF7L2 can assist in identifying individuals who may be less likely to benefit from GLP-1 RA monotherapy in a biomarker-driven tailored strategy. To improve glycemic and renal outcomes, those with these risk alleles may be eligible for alternative or combination therapy (such as SGLT2i) [115]. TCF7L2 genotyping has the potential to be used in clinical practice to customize diabetes therapy according to a patient’s genetic profile, but more investigation and confirmation are needed.

**Figure 6:**
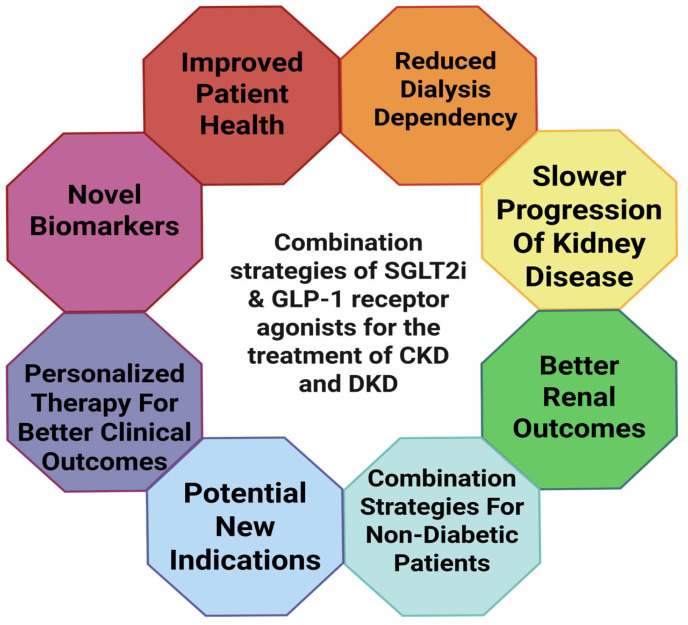
This figure outlines emerging areas of research, such as novel biomarkers, personalized therapy approaches, and potential new indications, in combination strategies of SGLT 2i and GLP-1 agonists for the treatment of CKD and DKD.

Emerging perspectives in the combined application of SGLT2i and GLP-1 RAs for CKD and DKD, highlighting advances in biomarker discovery, personalized treatment strategies, and novel therapeutic indications are depicted in Figure 6.

### 8.3. Health Equity Considerations

Promising clinical benefits, including better glucose control, fewer cardiovascular events, and renoprotection, have been demonstrated by combination therapy employing SGLT2i and GLP-1 RAs; however, these advantages must be weighed against their high costs, particularly in low-income settings. Here is a summary that takes cost-effectiveness and health fairness into account [116].

#### 8.3.1. Enhanced Clinical Outcomes

The combination makes use of complimentary mechanisms: GLP-1 RAs enhance glycemic control and aid in weight loss, while SGLT2i lower intraglomerular pressure and provide cardiovascular and renal protection. This collaboration has the potential to cut long-term healthcare expenses by reducing problems (such as hospitalizations and the advancement of renal disease).

#### 8.3.2. Cost-Effectiveness Considerations

According to economic assessments, SGLT2i and GLP-1 agonists may save money overall, even though their initial prices are higher since they can avoid expensive issues. For example, lower hospitalization rates and postponed end-stage kidney disease development can compensate for increased drug procurement expenses. These evaluations, however, frequently rely on data from high-income nations, and local pricing structures in low-income environments might vary greatly [117].

#### 8.3.3. Tiered Pricing and Negotiation

Government-led agreements and tiered pricing models can assist in reducing costs in low-income settings, improving access and making cost-effective medications more accessible to more patients.

#### 8.3.4. Inclusion in National Essential Medicines Lists

Including these combo treatments in programs for vital medications can help to lower costs by promoting local producers to create generic versions and facilitating subsidized access.

#### 8.3.5. Integrated Care Models

Combining cutting-edge treatments with effective chronic care and preventative care management in low-income environments can optimize clinical benefits while distributing expenses over a larger healthcare plan [117].

#### 8.3.6. Implementation Challenges

##### High Initial Costs

Both drug classes are considerably more expensive than traditional antidiabetic or antihypertensive agents. High costs limit accessibility, particularly in low- and middle-income countries, where the burden of CKD and diabetes is rising. Limited insurance coverage and out-of-pocket expenses often deter long-term adherence. Without sufficient subsidies, the absolute cost may still be too high for many healthcare systems and patients, even in cases when cost-effectiveness is proven [118].

##### Long-Term Safety Data

Although trials support the short-to-medium-term safety of these agents, comprehensive long-term data, especially beyond five years, are still lacking. Concerns remain about potential adverse events, such as

- SGLT2i: risks of genitourinary infections, ketoacidosis, and rare cases of Fournier’s gangrene.
- GLP-1 RAs: concerns about gastrointestinal intolerance, pancreatitis, and potential thyroid C-cell tumors (mainly in rodent studies) [119].

##### Infrastructure and Monitoring

Additional infrastructure and monitoring (such as laboratory testing and patient education) may be necessary for effective adoption, which can be difficult in environments with low resources. Initiation and titration of these therapies often require specialist oversight. GLP-1 RAs, especially injectable forms, may face resistance from patients due to their route of administration. Furthermore, variability in physician awareness and therapeutic inertia can delay optimal treatment decisions.

##### Need for Local Data

Localized cost-effectiveness analyses are essential to determine whether the long-term economic benefits observed in high-income countries are applicable in low-resource settings, considering diverse healthcare infrastructures and constraints.

Although the combination of GLP-1 RAs and SGLT2i has several clinical benefits that may eventually become cost-effective due to fewer problems, their high initial prices continue to be a substantial obstacle in low-income settings. To guarantee that the advantages of these treatments are available to everyone, regardless of financial situation, health equity issues necessitate customized approaches, such as tiered pricing, generic competition, and inclusion in national health programs [118].

#### 8.3.7. Telemedicine Protocols for Rural CKD Monitoring

By utilizing digital technology and local healthcare support, telemedicine guidelines for monitoring CKD in remote areas seek to close the gap in specialist care and enhance long-term results. The following essential elements are typically included in these protocols, which were created with cost-effectiveness and health equity in mind:

##### Remote Patient Monitoring

For efficient data collection, portable, validated instruments are used to measure important kidney parameters, such eGFR, blood pressure, weight, and albuminuria, at nearby clinics or through home monitoring. Real-time data must be transmitted to central healthcare systems through integration with telehealth platforms or smartphone apps.

##### Structured Virtual Consultations or Teleconsultations

Scheduled virtual consultations with multidisciplinary teams or nephrologists to discuss clinical data, modify medication regimens, and provide patient education. Tools for securing video conferences that guarantee privacy and excellent communication.

##### Integration with Local Healthcare Infrastructure

Community Health Workers (CHWs) are being trained to help patients with initial triage, data collection, and device usage by setting up telemedicine kiosks at remote clinics to offer access to telehealth services and technical assistance [116,119].

##### Standardized Protocols and Clinical Decision Support

Methods must be clearly defined for determining when to escalate therapy in response to worsening albuminuria, blood pressure spikes, or changes in eGFR slopes and incorporating decision-support tools to assist in deciphering data from remote monitoring and directing prompt actions.

##### Cost-Effectiveness and Health Equity

Scalable and low-cost technologies must be adopted and public or private finance must be used to offset the cost of connectivity and equipment. Regulation of telemedicine services should be incorporated into national insurance plans and healthcare programs, guaranteeing that access is not restricted by cost.

##### Incorporation of Patient Feedback

Telemedicine protocols for rural CKD monitoring can overcome resource and geographic constraints by concentrating on these factors, guaranteeing patients in low-income settings timely, affordable, and equitable care. This strategy not only improves CKD early identification and intervention but also lessens the burden of comorbidities, which eventually leads to better long-term health results [8,120].

## 9. Conclusions

The emergence of SGLT2i and GLP-1 RAs has transformed the management of CKD and DKD—shifting the paradigm from glucose-centric care to organ-protective, cardiorenal-metabolic strategies. These agents offer complementary benefits: SGLT2i reduce glomerular hyperfiltration, albuminuria, and inflammation, while GLP-1 RAs provide metabolic regulation, cardiovascular protection, and additional renal support. When combined, they offer a synergistic effect, addressing overlapping mechanisms of DKD pathogenesis and enhancing nephroprotection beyond monotherapy. Clinical trials and real-world data suggest that this dual approach improves both renal and cardiovascular outcomes, particularly in high-risk patients.

Their growing inclusion in treatment guidelines highlights a shift toward holistic, evidence-based CKD care. As ongoing studies assess long-term benefits, combination therapy is poised to become a cornerstone in preventing renal decline and cardiovascular morbidity in type 2 diabetes. Nevertheless, further research is needed to refine treatment protocols, identify ideal candidates, and ensure long-term safety and effectiveness.

## Figures and Tables

**Table 1:** Overview of Key SGLT2i approved for the Management of CKD (CKD) and DKD (DKD).

Name of Drug	Approval Status	Protein Binding and BA	Tmax and Half-Life	Cmax	Approval Indications
Tofogliflozin (10 mg) 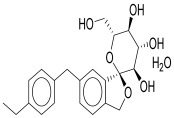	Approved in Japan in March 2014	83% and 97.50%	0.75 and 6.8	489 ng/mL	Highly selective SGLT2 inhibitor; moderate urinary glucose excretion
Ertugliflozin (5–15 mg once daily) 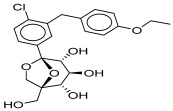	Approved in December 2017	95% and 70–90%	0.5–1.5 and 11–17	268 ng/mL (15 mg dose)	Type 2 Diabetes (DKD), Selective SGLT2 inhibition, promotes glycosuria and natriuresis
Ipragliflozin (50 mg once daily) 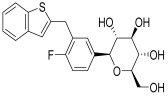	Approved in Japan January 2014 with	90% and 96.30%	1 and 15–16 (50 mg dose)	975 ng/mL	SGLT2 inhibition with low SGLT1 affinity; modest renal protection
Canagliflozin (100–300 mg once daily) 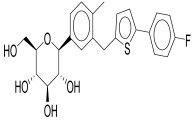	First SGLT2 inhibitor Approved in March 2013 (USA, Canada, Japan)	96% and 65% (300 mg dose)	1–2 and 10.6 (100 mg dose); 13.1 (300 mg dose)	1096 ng/mL (100 mg dose); 3480 ng/mL (300 mg dose)	Type 2 Diabetes, CKD with albuminuria
Empagliflozin (10 mg once daily) 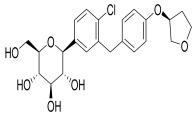	Approved in August 2014 with	86.20% and 90–97% (mice); 89% (dogs); 31% (rats)	1.5 and 13.2 (10 mg dose); 13.3 h (25 mg dose)	259 nmol/L (10 mg dose); 687 nmol/L (25 mg dose)	Type 2 Diabetes, Heart Failure, CKD (incl. DKD)
Luseogliflozin (2.5–5 mg once daily) 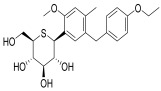	Approved in Japan in March 2014 with	96% and 35.3% (male rats); 58.2% (female rats); 92.7% (male dogs)	0.625 ± 0.354 and 9.24 ± 0.928	119 ± 27.0 ng/mL	Potent and selective SGLT2 inhibitor; shown to reduce albuminuria in early-stage DKD
Dapagliflozin (10 mg once daily) 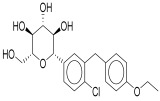	Approved by the EU in 2012	91% and 78%	1–1.5 and 12.9	79.6 ng/mL (5 mg dose); 165.0 ng/mL (10 mg dose)	Type 2 Diabetes, Heart Failure, CKD (incl. DKD)
Sotagliflozin (200–400 mg once daily) 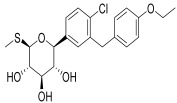	Approved in EU for Type 1 DM, under review in USA for CKD	~98%, Moderate; affected by food	13–20	~1200–1400 ng/mL	Type 1 Diabetes (Europe); Investigational for CKD

**Table 2:** Major Randomized Controlled Trials Demonstrating the Renoprotective Effects of SGLT2i in CKD and DKD Patients.

Trial	Year	Treatment or Drug	Study Population	Primary or Secondary End-Point	Key Findings
EMPA-REG OUTCOME	2015	Empagliflozin	CKD with or without T2DM (eGFR 20–45, or 45–90 with UACR ≥ 200)	Secondary	28% decrease in renal disease progression; benefit across broad CKD population
CANVAS	2017	Canagliflozin	T2DM + high CV risk; renal subgroup analyzed	Secondary	A persistent 40% decline in eGFR, slowed albuminuria progression; trend toward renal protection
CREDENCE	2019	Canagliflozin	T2DM + CKD (eGFR 30–90, UACR ≥ 300 mg/g)	Primary	Serum creatinine levels doubling, end-stage kidney disease, 30% reduction in primary composite outcome; renal and CV protection confirmed
DECLARE-TIMI	2019	Dapagliflozin	T2DM with/without CV disease (renal subcohort)	Secondary	47% in renal composite (sustained eGFR decline, ESRD, renal death); reduced albuminuria progression
DAPA-CKD	2020	Dapagliflozin	CKD with or without T2DM (eGFR 25–75, UACR 200–5000 mg/g)	Primary	Long-term eGFR decline of at least 50%, 39% decrease in primary endpoint; efficacy seen regardless of diabetes status
EMPEROR-Reduced	2020	Empagliflozin	Patients with eGFR ≥ 20 (regardless of diabetes status)	Secondary	Sustained eGFR < 10–15 mL/min/1.73 m^2^ or a sustained ≥40% decrease in eGFR and fewer renal events in empagliflozin group
EMPA-KIDNEY	2022	Empagliflozin	CKD with or without T2DM (eGFR 20–45, or 45–90 with UACR ≥ 200)	Primary	End-stage renal disease, a persistent drop in eGFR to less than 10 mL/min/1.73 m^2^, renal mortality, or a persistent eGFR decline of at least 40%, 28% reduction in renal disease progression
EMPEROR-Preserved	2021	Empagliflozin	Patients with/without T2DM and CKD	Secondary	Reduced HF hospitalization; renal benefits consistent in CKD subgroups

CANVAS (Canagliflozin Cardiovascular Assessment Study); CREDENCE (Canagliflozin and Renal Events in Diabetes with Established Nephropathy Clinical Evaluation); DAPA-CKD (Dapagliflozin in patients with CKD); DECLARE-TIMI (Dapagliflozin effect on cardiovascular events); eGFR (estimated glomerular filtration Rate); EMPA-KIDNEY (study of heart and kidney protection with empagliflozin); EMPA-REG OUTCOME (empagliflozin cardiovascular outcome event trial in type 2 diabetes mellitus patients); EMPEROR-Reduced (Empagliflozin Outcome Trial in patients with chronic heart failure with reduced ejection fraction).

**Table 3:** Comparative Profile of GLP-1 RAs: Dosing, Pharmacokinetics, Mechanism of Action, and Renal Safety Considerations in CKD/DKD Treatment.

Drug and Brand Name	Backbone	Dosage	Renal Dose Adjustment	Route of Elimination	Half-Life	Action
Long-Acting Compound						
Albiglutide	Human GLP-1	30 mg, 50 mg Once weekly, SC	Caution advised in moderate-severe renal impairment	Not available	~5 day	It mimics glucagon-like peptide-1 (GLP-1), stimulating insulin release and lowering blood sugar.
Dulaglutide	Human GLP-1	0.75 mg, 1.5 mg Once weekly, SC	Safe in CKD up to eGFR ~15; limited data in ESRD	Proteolytic degradation	~5 day	Safe and effective option for glycemic control in people with type 2 diabetes and moderate to severe CKD (CKD).
Semaglutide	Human GLP-1	0.5 mg, 1.0 mg Once weekly, SC	Effective across CKD stages; renal benefits seen in trials	Proteolysis; excreted via urine and feces	~1 wk	Semaglutide reduced the risk of major kidney disease events by 24% compared to placebo.
Exenatide ER	Exendin-4	2 mg Once weekly, SC	Not recommended for patients with an eGFR < 45 mL/min/1.73 m^2^ or ESRD	Glomerular filtration followed by proteolysis; eliminated in the urine	~1 wk	Extended-release GLP-1 RA; derived from GLP-1 RA, stimulates insulin secretion and delays gastric emptying.
Liraglutide	Human GLP-1	0.6–3 mg Once daily, SC	No adjustment in mild-moderate CKD; monitor in severe CKD	Proteolysis; excreted via urine and feces	~13 h	Short-acting GLP-1 RA; suppresses postprandial glucose via slowed gastric emptying and enhanced insulin response.
Liraglutide	Human GLP-1	0.6–1.8 mg Once daily, SC	No dosage adjustment required; not recommended for patients with CrCl < 15 mL/min	Proteolysis; excreted via urine and feces	~13 h	Activates GLP-1 receptors enhances insulin secretion, suppresses glucagon, promotes satiety.
Short-Acting Compound						
Exenatide	Exendin-4	5 μg, 10 μg Twice daily, SC	Avoid in severe CKD (eGFR < 30); risk of accumulation	Glomerular filtration followed by proteolysis; eliminated in the urine	~2.4 h	Activates GLP-1 receptors located on pancreatic β-cells, stimulates insulin secretion, suppression of glucagon secretion.
Lixisenatide	Exendin-4	10 μg, 20 μg Once daily, SC	Not recommended if eGFR < 30mL/min/1.73 m^2^	Glomerular filtration and proteolysis; excreted in the urine	~3 h	Short-acting GLP-1 RA; suppresses postprandial glucose via slowed gastric emptying and enhanced insulin response.
Oral Agent						
Semaglutide	Human GLP-1	3 mg, 7 mg, 14 mg Once daily	No dosage adjustment required	Proteolysis; excreted via urine and feces	~1 wk	Potent GLP-1 RA; reduces renal hyperfiltration, improves glycemic and CV markers; anti-inflammatory renal effects.
Fixed-Dose Combination						
Lixisenatide + glargine	Exendin-4	20 μg/iGlar 40 IU, 20 μg/iGlar 60 IU Once daily, SC	Closely monitor patients with CrCl 15–30 mL/min; not recommended for patients with CrCl < 15 mL/min	Glomerular filtration and proteolysis; excreted in the urine	~3 h	Mimics endogenous GLP-1, activating GLP-1 receptors on pancreatic β-cells, Suppresses glucagon secretion, Improved β-cell function and insulin sensitivity.
Liraglutide + degludec	Human GLP-1	1.8 mg/iDeg 50 IU Once daily, SC	Not studied in severe renal impairment; liraglutide is not recommended for patients with CrCl < 15 mL/min	excreted via urine and feces	~13 h	Binds to GLP-1 receptors on pancreatic β-cells, increases glucose-dependent insulin secretion, improve renal hemodynamics, Lower risk of hypoglycemia and weight gain, reduced insulin dose requirement.

**Table 4:** Summary of Landmark Clinical Trials Evaluating the Efficacy and Safety of GLP-1 RAs in Patients with Type 2 Diabetes and Associated Renal Outcomes in CKD.

Trial	Drug	Renal End Point	Results
AWARD-7	Dulaglutide versus glargine	Secondary	No significant lowering in urinary albumin-to-creatinine ratio (UACR); dulaglutide was more effective than insulin glargine in slowing renal function decline; changes in creatinine, cystatin C, and body weight did not show significant correlation.
LIRA-RENAL	Liraglutide versus placebo	Changes in eGFR Changes in UACR	Liraglutide and a placebo did not vary in terms of eGFR or UACR.
REWIND (NCT01394952)	Dulaglutide versus placebo	≥30% eGFR decline from baseline, ESRD	Dulaglutide decreases incidence of macroalbuminuria, eGFR decline ≥ 30%, considerably lowers the worsening eGFR. Need for dialysis 15%, decrease in (macroalbuminuria).
LEADER (NCT01394952)	Liraglutide versus placebo	Secondary 22% decrease in composite renal outcome (new macroalbuminuria, sustained eGFR decline, ESRD)	Liraglutide had a positive effect on macroalbuminuria; decreased incidence of nephropathy, patients with moderate-to-severe CKD saw a slower rate of reduction in eGFR over time.
EXSCEL	Exenatide LAR versus placebo	40% eGFR decline, Need for dialysis. Death for renal causes Macroalbuminuria	Exenatide LAR is more effective in reducing the occurrence of macroalbuminuria and performs better overall in terms of the composite renal outcome.
FLOW trial/Sustain-6 (NCT01394952)	Semaglutide versus placebo	(eGFR decline ≥ 50% from baseline, requirement for dialysis,	The actual role of GLP-1RAs as drugs that can stop type 2 diabetes patients’ disease from getting worse (driven by reduction in new-onset macroalbuminuria), 36% decrease in new or worsening nephropathy.
ELIXA	Lixisenatide versus placebo	Changes in UACR	Lixisenatide, independent of basal albuminuria, reduces the progression of UACR over time. There was no difference in the rate of eGFR reduction.
SCALE	Liraglutide versus placebo	Changes in UACR	In addition to a significant drop in weight, both liraglutide groups showed a lower urine albumin/creatinine ratio (UACR) as compared to the placebo group.
PIONEER 6	Oral semaglutide	Reduce albuminuria and slow eGFR decline	Supports the cardiovascular safety profile of oral semaglutide, PIONEER 6 provides important evidence that oral semaglutide can be safely used in patients with type 2 diabetes that are at high risk for cardiovascular events.
HARMONY	Albiglutide	Reducing cardiovascular risk	Provided strong evidence that GLP-1 RAs can reduce cardiovascular events in high-risk patients, reinforcing their role with proven cardioprotective benefits.
AMPLITUDE-O	Efpeglenatide	Sustained decline in eGFR, progression to end-stage renal disease, or renal death	Robust evidence showed efpeglenatide’s dual role in improving cardiovascular and renal outcomes, particularly among patients with coexisting cardiorenal comorbidities.

**Table 5:** Comparative overview of sodium–glucose cotransporter-2 inhibitors (SGLT2i) and glucagon-like peptide-1 receptor agonists (GLP-1 RA) across key clinical domains.

Mechanistic	SGLT2i	GLP-1RA
Primary Mechanism	Hemodynamic effects decrease glomerular hyperfiltration by preventing the reabsorption of glucose and sodium in the kidney’s proximal tubule. Hemodynamic changes, specifically natriuresis, volume depletion and intraglomerular pressure reduction, are the main drivers of their renoprotective implications.	Anti-inflammatory effects, lowering blood sugar and weight, GLP-1 receptor agonists can also reduce urinary albumin excretion by lowering circulating levels of proinflammatory cytokines and oxidative stress markers, improving endothelial function, and reducing inflammation in the glomeruli.
Clinical Implication	Slower progression of DKD and a lower chance of estimated glomerular filtration rate (eGFR) or progression to end-stage kidney disease (ESKD) are the results of lower blood pressure and glomerular pressure.	Significant decreases in albuminuria; these reductions are believed to be caused by the anti-inflammatory and anti-oxidative properties of these medications.
Efficacy in Key Populations (Diabetic CKD)	Reduce albuminuria and delay the deterioration of kidney function in diabetic CKD. Due to their hemodynamic effects, it lowers challenges of renal endpoints in diabetic patients.	Reduce albuminuria in diabetic populations, effect on hard renal endpoints including a substantial drop in eGFR or the development of ESKD.
Efficacy in Key Populations (Non-Diabetic CKD)	The effect in CKD patients without diabetes implicated through clinical trials like DAPA CKD. In both diabetic and non-diabetic CKD, these drugs lower progression to ESKD and prolonged fall in eGFR (independent of glycemic management).	Due to a lack of data and a less obvious molecular explanation in the absence of hyperglycemia, the role of GLP-1 receptor agonists in non-diabetic CKD is still unclear.
In Early CKD (Higher eGFR, Minimal Structural Damage)	In early CKD patients, SGLT2i promote a protective hemodynamic shift, reflected by a transient dip in eGFR which correlates with reduced albuminuria and slower disease progression.	In early CKD patients, GLP-1 RAs may modestly lower albuminuria by improving vascular health through endothelial function and reducing oxidative stress, though their influence on significant declines in eGFR remains limited.
In Advanced CKD (Lower eGFR, Greater Structural Damage)	In patients with advanced CKD, SGLT2i retain robust reno-cardiovascular benefits despite attenuated glycemic efficacy, as demonstrated by landmark trials, such as DAPA-CKD and EMPA-KIDNEY, supporting their therapeutic role beyond glucose control.	In advanced CKD, the renoprotective efficacy of GLP-1 RAs remains uncertain due to limited trial data; although they continue to be valuable for glycemic control and cardiovascular risk reduction.
MACE Reduction	Trials like EMPA REG OUTCOME have demonstrated that in CKD patients with established atherosclerotic disease, SGLT2i demonstrate a consistent reduction in MACE, though this effect is more modest in lower-risk populations.	In CKD patients, GLP-1 RAs offer significant cardiovascular protection, with consistent MACE reduction particularly in those with established cardiovascular disease, largely driven by their anti-inflammatory and anti-atherosclerotic effects (outcomes of LEADER, SUSTAIN6 and REWIND trials).
Heart Fail Divergence	In CKD patients, SGLT2i significantly reduce heart failure hospitalization and cardiovascular death through hemodynamic and neurohormonal mechanisms that alleviate myocardial stress and volume overload, independent of glycemic control (Outcome of Trial DAPA HF and EMPEROR Reduced).	In CKD patients, GLP-1 RAs demonstrate significant cardiovascular benefits, primarily by improving metabolic and inflammatory profiles, but their impact on heart failure progression remains minimal or neutral (Data from LEADER and SUSTAIN 6).
Safety and Tolerability	SGLT2i have a strong safety profile in CKD patients, with only a slight increase in risk for vaginal and urinary tract infections. As oral agents, they avoid injection-related issues and are generally well tolerated, with manageable side effects like volume depletion and polyuria.	GLP-1 RAs commonly cause dose-dependent gastrointestinal side effects and injection-site reactions, which are generally mild and transient. Severe adverse events are rare, and the risk of hypoglycemia remains low when not combined with insulin secretagogues.

**Table 6:** Comparative summary of major clinical trials evaluating GLP-1 RAs and SGLT2i in patients with CKD and DKD.

Category	Drug	Clinical Trails
SGLT2i trials	Empagliflozin	•EMPA-REG OUTCOME: 28% reduction in renal disease progression; benefit across broad CKD spectrum
		•EMPEROR-Reduced: Reduction in sustained ≥40% eGFR decline or progression to renal failure
		•EMPA-KIDNEY: 28% reduction in renal disease progression; increased ESKD, eGFR decline ≥40%, renal death
		•EMPEROR-Preserved: Renal benefit consistent in CKD subgroup; not a primary renal trial
	Canagliflozin	•CANVAS: Slowed albuminuria progression; trend toward renal protection (secondary endpoint)
		•CREDENCE: 30% reduction in primary renal outcome (ESKD, doubling of creatinine, renal death)
	Dapagliflozin	•DECLARE-TIMI: 47% reduction in renal composite (sustained eGFR decline, ESRD, renal death); reduced albuminuria
		•DAPA-CKD: 39% reduction in primary renal endpoint; benefits seen with/without diabetes
GLP-1 RA Trials	Dulaglutide	•AWARD-7: Slowed eGFR decline vs. insulin glargine; no significant UACR change
		•REWIND: Reduced Macroalbuminuria; reduce ≥30% eGFR decline; 15% reduction in dialysis need
	Liraglutide	•LIRA-RENAL: No significant difference in eGFR or UACR vs. placebo
		•LEADER: 22% reduced composite renal outcome (macroalbuminuria, sustained eGFR decline, ESRD)
		•SCALE: Reduced UACR with weight loss benefit
	Exenatide LAR	•EXSCEL: Reduced Macroalbuminuria; improved composite renal outcomes
	Semaglutide	•SUSTAIN-6/FLOW: 36% reduction in new/worsening nephropathy; Decreased macroalbuminuria
		•PIONEER 6: Reduced Albuminuria; slowed eGFR decline; safe in high CV risk diabetic population
	Lixisenatide	•ELIXA: Reduced UACR progression; no change in eGFR
	Albiglutide	•HARMONY: CV benefit shown; renal outcomes not directly reported
	Efpeglenatide	•AMPLITUDE-O: Reduced Sustained eGFR decline, ESKD, and renal death

## Data Availability

Not applicable.
